# Unrestricted Versus Restricted Anterior Knee Displacement During Bodyweight Squat Training in Physically Active Young Adults: An Exploratory Three-Arm Randomized Controlled Trial

**DOI:** 10.3390/jcm15187162

**Published:** 2026-09-15

**Authors:** Amirkiya Movahhedi, Adem Çalı, Ömer Şevgin

**Affiliations:** 1 Department of Physiotherapy and Rehabilitation, Graduate School, Bahçeşehir University, Istanbul 34734, Türkiye; fztamirkiya@gmail.com; 2 Department of Physiotherapy and Rehabilitation, Faculty of Health Sciences, İstanbul Topkapı University, Istanbul 34087, Türkiye; 3 Department of Physiotherapy and Rehabilitation, Faculty of Health Sciences, Üsküdar University, Istanbul 34768, Türkiye; omer.sevgin@uskudar.edu.tr

**Keywords:** squat, anterior knee displacement, knees over toes, exercise technique, randomized controlled trial, Oswestry Disability Index, pressure pain threshold, Y Balance Test, vertical jump

## Abstract

**Background/Objectives:** Restricting anterior knee displacement is a common squat cue, but it redistributes rather than reduces mechanical demand, and comparative evidence is largely acute. This exploratory trial compared the short-term functional effects in healthy, active young adults. **Methods:** Sixty participants were randomized to unrestricted or restricted anterior knee displacement, or usual activity (three-arm, single-centre). Both exercise groups completed 18 remotely supervised bodyweight squat sessions over six weeks (720 repetitions; 100% session adherence among completers). The primary outcome was low-back-related disability (Oswestry Disability Index); 21 exploratory secondary outcomes covered pressure pain threshold, Y Balance Test reach, exertion, and jump flight time. Follow-up scores were compared by one-way analysis of variance, unadjusted for baseline, with post hoc ANCOVA sensitivity analyses as supplementary. **Results:** Fifty-seven of the 60 randomized participants completed the trial and were analysed (*n* = 19, 18, and 20). Follow-up Oswestry scores did not differ among groups, F(2,54) = 1.999, *p* = 0.145, η^2^ = 0.069; the largest pairwise difference was −2.9 percentage points (95% CI −5.9 to 0.0). Baseline disability was minimal (floor effect). In the unadjusted analysis, only the left-hamstring pressure pain threshold reached *p* < 0.05, not surviving multiplicity adjustment; baseline-adjusted sensitivity analyses left the primary result unchanged while strengthening several exercise-versus-control contrasts, two below the adjusted threshold—all exploratory. Comparable changes occurred in the untreated control group. **Conclusions:** Within the conditions of this short-term bodyweight-squat intervention, restricting anterior knee displacement provided no detectable advantage, and no clinically important between-group difference. The trial was not an equivalence study.

## 1. Introduction

The squat is a fundamental closed-chain, multi-joint exercise used across rehabilitation, general fitness, and athletic preparation. Its apparent simplicity conceals a coordinated interaction among the ankle, knee, hip, pelvis, and trunk, and changes in any one segment can redistribute motion and loading throughout the kinetic chain [1,2]. Squat technique is therefore commonly modified to target specific adaptations, accommodate mobility constraints, or manage symptoms.

One of the most persistent technical debates concerns anterior knee displacement relative to the toes. The instruction to keep the knees behind the toes is commonly justified as a means of reducing knee loading. Acute biomechanical studies, however, show that restricting forward knee travel does not simply remove load; it reduces knee torque while increasing the mechanical demand placed on the hip and trunk [3,4,5,6]. A contemporary review concluded that some anterior knee displacement is often necessary to maintain the centre of mass over the base of support and that a universal prohibition is difficult to justify in healthy individuals [7]. Conversely, permitting greater anterior knee displacement can increase patellofemoral reaction force and stress under some conditions, supporting context-specific restriction in selected rehabilitation settings [8].

The expression of anterior knee displacement is also shaped by the wider movement strategy. Stance width, foot-rotation angle, training experience, and body position alter knee, hip, and trunk kinematics and kinetics during squatting [9]. Movement speed and support-surface stability can modify lower-limb muscle activation and postural demands [10]. Consequently, the position of the knees relative to the toes cannot be interpreted independently of depth, trunk inclination, ankle dorsiflexion, external load, cadence, anthropometry, and the purpose of the exercise.

Despite extensive biomechanical investigation, most evidence on restricted and unrestricted anterior knee displacement is derived from acute laboratory comparisons rather than longitudinal training studies [3,4,5,6,7,8]. It therefore remains uncertain whether repeatedly practicing these patterns produces measurably different adaptations. Acute differences in joint moments do not necessarily translate into differences in pain sensitivity, balance, patient-reported function, or performance after several weeks of training.

Dynamic balance and vertical jumping represent distinct neuromuscular demands. Dynamic balance requires unilateral postural control, reach coordination, strength, and mobility, whereas vertical jumping depends on rapid force production and intersegmental coordination. An eight-week randomized bodyweight neuromuscular program has improved dynamic balance and vertical-jump performance in trained young athletes [11], but the transfer from slow bilateral squatting to multidirectional balance and ballistic jumping may be limited by task specificity.

The novelty of the present study lies in extending the knees-over-toes question beyond acute kinematic and kinetic comparisons to a repeated, remotely supervised bodyweight training intervention. It combines low-back-related disability, pressure pain threshold, multidirectional dynamic balance, perceived exertion, and vertical-jump performance within a three-arm design that includes a usual-activity control group. The control arm is particularly relevant because repeated performance testing can be influenced by familiarization and test–retest variability. The primary objective was to determine whether 18 sessions of bodyweight squatting performed with unrestricted or restricted anterior knee displacement produced a greater reduction in low-back-related disability, measured with the Oswestry Disability Index, than the alternative pattern or usual activity. We acknowledge at the outset that this endpoint is poorly matched to an asymptomatic, physically active population, in which the instrument has limited capacity to register change; the analysis is retained for consistency with the registration, but the trial is interpreted throughout as an exploratory comparison of the short-term functional effects of two squat strategies rather than as a test of efficacy for disability reduction. Secondary objectives were to compare the same three conditions across pressure pain threshold, dynamic balance, perceived exertion, and vertical-jump flight time. No directional hypothesis and no equivalence or non-inferiority margin were specified for either objective.

## 2. Materials and Methods

### 2.1. Study Design, Setting, and Reporting

This was a single-centre, three-arm, parallel-group randomized controlled trial in healthy, physically active adults. Volunteers were recruited by open announcement within the university community, using notice boards, institutional e-mail, and word of mouth, and were enrolled consecutively as they presented; no other recruitment route was used. All face-to-face baseline and post-intervention assessments were carried out in the physiotherapy assessment laboratory of Bahçeşehir University, Istanbul, Türkiye, while the exercise sessions themselves were delivered by telerehabilitation to participants at home. Recruitment, intervention, and follow-up assessment took place between March 2025 and May 2025, and the trial ended as planned once the target sample had completed the protocol. The aim, scope, assessment procedures, and intervention protocols were explained to each volunteer using an informed-consent form, and written and verbal consent were obtained before participation. The study was approved by the Non-Interventional Research Ethics Committee of Üsküdar University (protocol code 61351342/020-1245, decision no. 12, approved 29 December 2024). The trial record was submitted to ClinicalTrials.gov (NCT06843785) on 19 February 2025 and first posted on 25 February 2025; both dates precede enrolment of the first participant on 1 March 2025, so registration was prospective. The two dates are stated together here because earlier and current versions of this manuscript cited them inconsistently. No patients or members of the public were involved in the design, conduct, analysis, or reporting of this trial.

Reporting follows the CONSORT 2025 statement for randomized trials [12] together with the CONSORT extension for non-pharmacological treatments [13], and a completed CONSORT 2025 checklist and participant-flow diagram are provided as Supplementary Material, replacing the CONSORT 2010 checklist submitted with the original manuscript. The statement in the previous version that no changes were made to the methods or outcomes after trial commencement was inaccurate and has been withdrawn. Several differences between the original registry record and this report are declared here in full, and an amendment correcting the entries identified below as errors was submitted to the registry in August 2026. ClinicalTrials.gov posts amendments only after quality-control review, so the publicly displayed record may still show the previous version while this manuscript is under review; the declarations in this paragraph, not the state of the public record on any given date, should be taken as describing the trial. The original February 2025 version remains available in the ClinicalTrials.gov archive and is provided as Supplementary File S1. First, the registration described the trial as triple masked; this entry was made in error, because the interventions involve visibly different movement instructions and no party was in fact blinded (Section 2.4). Second, the registry referred to computerized randomization with a closed-envelope method; envelopes were not used, and the procedure actually followed is described in Section 2.4. Third, the registry contained inconsistent statements of intervention duration, referring in the same paragraph to both four and six weeks; the protocol delivered was 18 supervised sessions performed three times per week over six weeks (Section 2.5). Fourth, muscle thickness and muscle strength appeared in the registry description but were never measured and are therefore not reported. Fifth, the registry described squat depth as being measured with a ruler and goniometer for further analysis; depth was in fact checked during the instructional visit as a technique-teaching aid, and the values were not retained as analysed variables (Section 2.5). Sixth, the registry described a fixed schedule of one set of 20 repetitions per session; the delivered schedule was progressive, from 1 set of 20 to 2 sets of 30 repetitions, as described in Section 2.5, while the session frequency and duration (three sessions per week for six weeks, 18 sessions in total) were as registered.

One further difference concerns the classification of outcomes and requires explicit statement. The original registration listed three co-primary outcome measures—the Oswestry Disability Index, the Y Balance Test, and the IPAQ-SF—and listed no secondary outcomes. In the present report the Oswestry Disability Index is treated as the primary outcome and all other measures, including the Y Balance Test, are treated as secondary; this reclassification was made at the analysis stage and was not prespecified. The IPAQ-SF, registered as a co-primary outcome, was used to establish eligibility and is reported only as a baseline characteristic (Section 2.2), not as an outcome. A Y Balance Test composite score was described in the registry but is not reported, because the reach directions were recorded and analysed individually; the composite can be derived from the values in Section 3.6. Conversely, pressure pain threshold, perceived exertion, and vertical-jump flight time are reported here although they were not entered in the registry outcome fields. These three measures were specified in the master’s-thesis protocol from which this trial derives, and the corresponding measurement forms are appendices to the thesis; they were, however, never entered in the registry outcome fields. Because their prespecification therefore cannot be demonstrated from the public registry record, they are interpreted as exploratory throughout this report. Self-reported knee function (IKDC) was likewise collected but is not reported in this version: during the present revision its scoring could not be verified against the original source records, and the outcome has been withdrawn rather than presented with unverifiable values (Section 2.6.3). All secondary outcomes, including those reclassified from the registry and those not registered, are interpreted as exploratory throughout. The registry outcome fields were revised in the same August 2026 amendment to correspond to the outcomes reported here; as noted above, that amendment may not yet be posted, so the public record may still display the original three co-primary outcomes. The original February 2025 version, which shows the outcome list as originally registered, is provided as Supplementary File S1 so that the two records can be compared directly, and the classification declared in this paragraph is the one that should be taken as describing what was prespecified. No amendment to the protocol was made after any examination of outcome data.

### 2.2. Participants

Eligible participants were 18–30 years old, classified as physically active using the International Physical Activity Questionnaire-Short Form (IPAQ-SF) [14,15], free of any psychological, neurological, orthopaedic, or rheumatological disorder, without lower-extremity fracture or surgery during the previous year, able to perform at least one conventional squat, and able to communicate in Turkish. All 60 enrolled participants met the active category of the IPAQ-SF; the questionnaire was administered to determine eligibility, and the numerical MET-min/week scores were not retained for analysis, so they cannot be reported as a baseline characteristic. Exclusion criteria were pregnancy or suspected pregnancy; diagnosed knee valgus, pes planus or pes cavus, or hip-rotation disorder; a self-reported or previously diagnosed interlimb strength deficit; a physician-diagnosed painful knee condition of recent onset (the previous version used the term “acute knee osteoarthritis”, which is not an appropriate diagnostic category in an 18–30-year-old population and has been corrected); regular medication use or use of non-steroidal anti-inflammatory or similar disease-modifying medication during the previous month; intra-articular hyaluronic acid or steroid injection during the previous 6 months; and a comorbidity contraindicating exercise, such as advanced osteoporosis, vertigo, or neurological disease. Eligibility was determined by clinical history and physical examination by the treating physiotherapist; no instrumented strength testing was performed, so no numerical interlimb asymmetry threshold was applied and this criterion was screened qualitatively. Ankle dorsiflexion range of motion, which is an anatomical determinant of attainable anterior knee travel and squat depth, was not measured at screening or at any subsequent time point; the groups therefore cannot be shown to have been comparable in their capacity to comply with the assigned movement pattern, and this is acknowledged as a limitation. Protocol-specified withdrawal criteria were participant request, exercise-related pain above 5/10 on a visual analogue scale, a traumatic injury affecting performance, or missing three consecutive sessions. Harms were assessed non-systematically rather than with a structured instrument: exercise-related pain was queried by the supervising physiotherapist during each synchronous session against the protocol-specified 5/10 visual-analogue-scale withdrawal threshold, and any spontaneously reported adverse event or injury was recorded. Because no symptom checklist was administered at fixed intervals, mild or transient events may have gone unrecorded.

### 2.3. Sample Size

A power analysis was performed to determine the minimum number of participants required. Test power was calculated with G*Power (version 3.1.9.7; Heinrich Heine University Düsseldorf, Düsseldorf, Germany) for a one-way analysis of variance. Based on previous functional-performance literature [16], an effect size of 0.799, a 5% type I error rate (α = 0.05), and 90% power (1 − β = 0.90) were assumed, yielding 20 participants per group and 60 in total. The study was therefore designed with 20 participants in each of the unrestricted, restricted, and control groups. This calculation has two consequences that constrain every inference drawn below. It was anchored to a functional-performance outcome rather than to the registered primary outcome, so no disability-specific effect size, variance estimate, or minimal important difference informed the target sample; and the assumed effect was large. With the 57 participants ultimately analysed, the smallest between-group effect detectable at 80% power was η^2^ ≈ 0.15. The trial was therefore powered to detect only large differences and cannot exclude small or moderate ones. No equivalence or non-inferiority margin was prespecified, and the design does not support equivalence or non-inferiority conclusions. No interim analysis was planned or performed, and no formal stopping guideline was specified.

### 2.4. Randomization, Allocation Concealment, and Blinding

Allocation used restricted randomization based on a pre-generated allocation list. An independent researcher not otherwise involved in the trial prepared a list containing 20 entries for each of the three groups and randomly permuted it using the random-number functions of Microsoft Excel (Microsoft Corp., Redmond, WA, USA; version not recorded; https://www.microsoft.com/microsoft-365/excel, accessed on 14 September 2026); because the list was of fixed composition, this procedure fixed the final group sizes at 20/20/20 and no blocking or stratification was required. Participants were screened for eligibility and enrolled by one investigator (A.M.) and were then assigned to the next allocation on the list by the same independent researcher, sequentially, as they presented. Because the allocation list was prepared and held by a person outside the research team and was not accessible to the investigator enrolling participants, the upcoming allocation was concealed at the point of enrolment; sequentially numbered, opaque, sealed envelopes were not used, and the registry description of a closed-envelope method was entered in error. Because the interventions involved visibly different movement instructions, neither participants nor the treating physiotherapist could be blinded to the assigned technique. Outcome assessors were also not blinded to group allocation, and the registry entry describing the trial as triple masked is therefore incorrect and has been declared in Section 2.1.

### 2.5. Interventions

Both exercise groups completed the same progressive 18-session bodyweight squat schedule under remote physiotherapist supervision, performed three times per week over six weeks. Sessions 1–3 comprised 1 set of 20 repetitions; sessions 4–6 and 7–9, 1 set of 30 repetitions; sessions 10–12, 2 sets of 20 repetitions; and sessions 13–15 and 16–18, 2 sets of 30 repetitions, giving a cumulative volume of 720 repetitions. Repetitions within a set were performed in alternating blocks of approximately 30 s of continuous squatting and 30 s of rest; because a metronome (make and model not recorded) set at 60 beats/min imposed approximately 2 s for descent, a 2 s hold at the bottom position, and 2 s for ascent (approximately 6 s per repetition), each 30 s work block contained approximately five repetitions, so a set of 20 repetitions comprised approximately four work–rest blocks and lasted approximately 4 min. This clarifies an internally inconsistent description in the previous version, in which the metronome cadence and the 30 s work–rest structure could not be reconciled. Participants stood with the feet approximately pelvis-width apart, toes turned outward by approximately 15–30°, arms extended forward, gaze directed forward, and heels maintained on the floor. A physiotherapist taught the technique in person at the baseline visit and supervised every session in real time by synchronous video call, providing verbal correction when position errors occurred. Perceived exertion was recorded verbally at the start and end of each session for monitoring purposes; the Modified Borg values reported in Section 3.7 are the baseline and post-intervention assessment values obtained at the two face-to-face visits, not session-level values (Section 2.6.3). All instructional visits and all supervised exercise sessions were delivered by a single qualified physiotherapist (A.M.); no other individual delivered the intervention or the comparator, and the trial was conducted at a single site. Years of clinical experience are not reported.

Because every session was delivered synchronously by video call, attendance was verified in real time by the supervising physiotherapist rather than by participant self-report. All 37 participants who completed the trial in the two exercise groups (unrestricted, *n* = 19; restricted, *n* = 18) attended all 18 prescribed sessions with no missed sessions, so adherence among completers was 100% and every completer performed the full cumulative volume of 720 repetitions. Here, 100% session adherence among completers means that no prescribed session was missed by any completer, as established in real time by the supervising physiotherapist during each synchronous video call. Session-level attendance records—dates of individual sessions and any rescheduling—were not, however, retained as analysable documents, so this figure rests on the real-time verification just described and cannot be re-derived from the study dataset; as with the algometry application rate, this limitation of the trial’s documentation is stated here directly (see also Section 4.6). Progression between blocks was fixed by the schedule above and was not conditional on individual performance criteria. Technique errors were corrected verbally as they occurred but were not counted, so their frequency cannot be quantified. Participants in all three groups were asked not to begin any new structured exercise programme during the study period, but habitual physical activity and additional exercise in the control group were not monitored quantitatively after baseline; this is acknowledged as a limitation, particularly because several outcomes changed within the untreated control group.

In the unrestricted anterior knee displacement group, participants were allowed to move the knees beyond the toes and squatted to their most comfortable depth. In the restricted anterior knee displacement group, a rigid wooden board of standard dimensions (60 cm wide) was placed on the floor immediately distal to the first toe of both feet, with its long axis perpendicular to the direction of gaze, so that it spanned the width of the stance and physically blocked forward translation of the knees; the same board was used for every participant in this group, and neither its dimensions nor its position was adjusted for stature, limb length, or foot size. Participants in this group were instructed to squat to approximately 90° of knee flexion. During the instructional visit, achieved depth was checked with a goniometer at the knee and with a ruler measuring the vertical hip-to-floor distance (the make and model of both instruments were not recorded); these measurements were used to teach and standardize the target position, and the values were not retained, so they cannot be reported. No kinematic manipulation check of anterior knee travel was performed during the training sessions themselves, and the contrast delivered is therefore best described as two squat-instruction packages rather than isolated restriction of anterior knee displacement. Contact between the knee and the board during supervised sessions was treated as a technique error: the repetition was verbally corrected in real time by the physiotherapist observing the synchronous video feed, repeated, and not counted toward the prescribed volume. The control group continued usual daily activities and did not receive the squat program.

### 2.6. Outcome Measures

The outcome designated as primary in this report is low-back-related disability, measured with the Turkish version of the Oswestry Disability Index. As set out in Section 2.1, the registration listed this measure together with the Y Balance Test and the IPAQ-SF as co-primary outcomes and listed no secondary outcomes; the classification used here was made at the analysis stage and was not prespecified. All remaining measures listed below are treated as secondary. Because 21 secondary outcomes were analysed without multiplicity correction, and because several of them were not entered in the registry outcome fields, all secondary results are interpreted as exploratory throughout.

#### 2.6.1. Pressure Algometry

Pressure pain threshold was assessed bilaterally at the L1–L3 paraspinal region, iliac region, gluteus medius, hamstrings, knee, and greater trochanter by a single trained researcher using a digital algometer with a 1 cm^2^ rubber tip (FDX 50; Wagner Instruments, Greenwich, CT, USA), which has published reliability and validity evidence [17,18]. The same researcher, who was aware of group allocation, performed all baseline and all post-intervention measurements. Paraspinal stimuli were applied 5 cm to the right and left of the L3 level. The device was set to a 1000 Hz sampling rate with a maximum-reading hold function and had a resolution of 0.2 N over a 250 N capacity. Pressure was applied perpendicular to the skin and increased gradually in accordance with the procedure described by Kinser and colleagues [17] until the participant reported the first sensation of pain, at which point the participant said “stop” and the value was recorded. The rate of pressure application was not instrumented, standardized, or recorded, so the actual application rate cannot be reported and is stated here directly as a limitation of the measurement procedure rather than described only as gradual. Three measurements were taken at each site and the mean of the three was used for analysis. Sites and sides were tested in the same fixed order for every participant; the order was not randomized, so a systematic order effect cannot be excluded. Values are reported in kgf/cm^2^. For participant safety, pressure application was terminated at 11 kgf/cm^2^ whether or not the pain threshold had been reached, so this value represents a protocol-imposed ceiling and observations at the ceiling are right-censored. The number of observations reaching the ceiling was not quantified, and because censoring violates the distributional assumptions of the parametric tests applied, pressure-pain-threshold results at the sites most affected by the ceiling—particularly the gluteus medius and greater trochanter, whose post-intervention means lay within 10% of the ceiling—should be interpreted with corresponding caution.

#### 2.6.2. Y Balance Test

Dynamic balance was assessed barefoot using the Y Balance Test. The test was performed on a set-up assembled in the laboratory rather than on a commercially manufactured kit, so no manufacturer information can be given. The instrumented Y Balance Test was developed to improve standardization of the Star Excursion Balance Test and has demonstrated acceptable reliability [19,20,21]. The tested limb was defined as the stance limb, and three reach directions were recorded for each stance limb: anterior, posteromedial, and posterolateral. These standard direction names replace the non-standard labels used in the previous version, in which reach direction was described relative to body side; reaches away from the midline are reported here as posterolateral and reaches across the midline as posteromedial. Limb length was measured with the participant supine as the distance from the most inferior aspect of the anterior superior iliac spine to the most distal aspect of the medial malleolus, to the nearest 0.1 cm. Participants completed six practice attempts for each limb and direction to minimize learning effects, followed by three recorded trials, with at least 10 s of rest between trials and at least 30 s between reach directions. A trial was discarded and repeated if the participant moved the stance foot from the platform or crossed the marked line, pushed or stepped on the reach indicator, touched down with the free foot, or lost balance before returning the free foot to the starting position. Reach distance was averaged across the three trials and recorded to the nearest centimetre. Correction (this revision): the analysed values are these raw reach distances in centimetres, not limb-length-normalized scores. The normalization described in previous versions—(reach distance/limb length) × 100—was planned but was not applied in the analysed dataset: all 684 Y Balance Test values in the dataset are integer centimetre distances, and the individual limb-length measurements, although summarized in Table 1 from the source records, were never entered into the electronic dataset, so normalized scores cannot be reconstructed. All Y Balance Test values in Section 3.6, in Tables S1 and S2, and Supplementary Data S1 are therefore unnormalized reach distances in centimetres; because mean limb length was similar across groups (Table 1), this affects the units and the comparability with normalized values in the literature, not the within-trial group contrasts. Two researchers, one of whom delivered the test instructions, recorded reach distances independently; inter-rater agreement was not quantified.

#### 2.6.3. Self-Reported Outcomes and Perceived Exertion

Low-back-related disability, the primary outcome, was assessed at baseline and after the intervention using the Turkish version of the Oswestry Disability Index [22]. Each of the ten items is scored 0–5, giving a raw total of 0–50, which is doubled to yield a percentage; scores of 0–20% denote minimal disability. Both the raw total and the percentage are reported. A change of approximately 10 percentage points is the most widely cited minimal clinically important difference for this instrument [23,24], and this value is used as the reference threshold throughout. Perceived exertion was rated on the 0–10 Modified Borg scale [25]. Ratings were collected in two distinct ways, which the previous version did not distinguish: session-level ratings were obtained verbally before and after each supervised exercise session in the two exercise groups only and were used for monitoring; and assessment-level ratings were obtained from all three groups, including the control group, at the baseline and post-intervention face-to-face visits, referring to perceived leg fatigue during the standardized test battery performed at that visit. Only the assessment-level ratings are reported in Section 3.7, which is why values are available for the control group even though it performed no exercise sessions. Knee symptoms and function were assessed at baseline and after the intervention using the Turkish version of the 2000 International Knee Documentation Committee (IKDC) subjective knee form [26]. During the present revision, however, the previously reported IKDC values (means of approximately 71 with standard deviations of 1.0–1.6 and a total observed range of 68–73) could not be verified against the original source records, and a scoring error could not be excluded. The IKDC outcome has therefore been withdrawn from this report in its entirety—from the outcome list, the outcome tables, and the Results and Discussion—rather than presented with unverifiable values. No other outcome is affected by this withdrawal. The participant-level dataset submitted with this revision (Supplementary Data S1) contains the IKDC entries exactly as recorded—integer values between 68 and 73 only—so the basis for the withdrawal can be inspected directly.

#### 2.6.4. Vertical Jump Testing

Squat jump (SJ) and countermovement jump (CMJ) were recorded with the My Jump 2 smartphone application (C. Balsalobre-Fernández, Madrid, Spain; version not recorded; https://apps.apple.com/us/app/my-jump-2/id1148617550, accessed 14 September 2026), with the smartphone positioned at floor level approximately 1.5 m from the participant. For SJ, participants began at approximately 90° of knee flexion and jumped maximally without a countermovement, avoiding trunk flexion; for CMJ, participants descended to approximately 90° of knee flexion and immediately jumped maximally. Hands remained on the hips throughout. Smartphone flight-time methods have demonstrated acceptable validity and reliability when standardized procedures are used [27,28]. Flight time in milliseconds was the jump metric specified in the thesis protocol and designated for analysis (it was not entered in the registry outcome fields; Section 2.1); the jump-height and power values derived by the application were not analysed, because they are algebraic transformations of flight time and add no independent information.

### 2.7. Statistical Analysis

Analyses were performed using IBM SPSS Statistics version 26 (IBM Corp., Armonk, NY, USA). The analysis set comprised the 57 participants who completed the trial and provided follow-up data. This is an available-case (complete-case) analysis and not a strict intention-to-treat analysis: three of the 60 randomized participants discontinued and contributed no follow-up measurements, no imputation was performed, and no sensitivity analysis for missing data was carried out. Continuous data were summarized as mean and standard deviation, and categorical data as counts. Normality was examined using the Shapiro–Wilk test. The primary analysis compared follow-up Oswestry Disability Index scores among the three groups using one-way analysis of variance at α = 0.05. The same approach was applied to each secondary outcome, with Tukey HSD post hoc tests, or Games–Howell tests where the homogeneity-of-variance assumption was not met. For every outcome, the between-group mean difference and its 95% confidence interval are now reported for all three pairwise contrasts (Section 3.4 and Table S1), because effect estimates with confidence intervals are more informative than significance testing alone. Sex and dominant-limb distributions were not compared statistically, because significance testing of baseline characteristics after randomization is uninformative; where categorical comparison is required, Fisher’s exact test rather than the chi-square test is appropriate given expected cell counts below five. Within-group pre-post comparisons used paired-samples *t* tests where the Shapiro–Wilk test on the within-group differences indicated normality (*p* ≥ 0.05) and Wilcoxon signed-rank tests otherwise; the test used is indicated for every comparison in the outcome tables (†). These comparisons are descriptive only and are not evidence of comparative efficacy. In response to peer review, all within-group *p* values and effect sizes were recomputed from the dataset under this single rule; the standardization changed several entries carried over from the source thesis, including the removal of previously reported significant control-group changes on the Modified Borg scale, at the right gluteus medius, and at the right greater trochanter (Section 3.5 and Section 3.7), and every changed entry is highlighted in the tables. Eta-squared was calculated as SS-between/SS-total, and Cohen’s d for within-group changes from between-subject standard deviations. No multiplicity correction was applied to the 21 secondary outcomes; a Bonferroni-adjusted threshold of *p* < 0.0024 is therefore used as a reference point when interpreting individual secondary results.

We acknowledge that this analysis strategy is not the strongest available. For a randomized trial with baseline and follow-up measurements, analysis of covariance with the follow-up score as the dependent variable, the baseline score as a covariate, and randomized group as a fixed factor—or a mixed model including group, time, and the group-by-time interaction—uses the available baseline information and generally provides a more precise and less biased estimate of the intervention effect than comparison of follow-up values alone. It is particularly preferable for left anterior Y Balance Test reach, which differed among groups at baseline. The analyses reported in the main text are those conducted in the master’s thesis from which this report derives, so all between-group differences reported in the main-text outcome tables and in Table S1 are unadjusted for baseline and should be read on that basis. In response to peer review, a post hoc baseline-adjusted sensitivity re-analysis—analysis of covariance with the follow-up score as the dependent variable, the baseline score as covariate, and randomized group as a fixed factor—was additionally performed for the primary outcome and all 21 secondary outcomes and is reported in full in Table S2 and summarized in Section 3.10. These sensitivity analyses were not prespecified. The one outcome with a demonstrable baseline imbalance, left anterior Y Balance Test reach, remains uninterpreted in the primary presentation; its baseline-adjusted estimate is provided in Table S2. For the present revision, every descriptive and inferential statistic in the main-text tables was additionally re-verified, cell by cell, against the participant-level dataset now submitted as Supplementary Data S1. This check identified three transcription errors in Table 2 (one baseline mean, one follow-up mean, and one follow-up standard deviation, all in the restricted group, with the two within-group effect sizes derived from them) and five errors in reported F or η^2^ values (four in Table 2, one in Table 3); all are corrected and marked in red, none changes any inference, and an item-by-item verification report documenting the check is provided as Supplementary File S2. The in-text values derived from the affected cells—the η^2^ range in Section 3.4 and the ceiling-proximity figures in Section 2.6.1 and Section 3.5—were corrected accordingly and are likewise marked in red. For the present resubmission, the correspondence between the source thesis, the dataset, and this report was additionally verified: all outcome statistics extractable from the thesis were reproduced from the dataset, and the three corrected Table 2 cells can be traced to transcription errors in the thesis tables rather than to the data (Supplementary File S2, Section S7).

## 3. Results

### 3.1. Participant Flow and Recruitment

**Figure 1:**
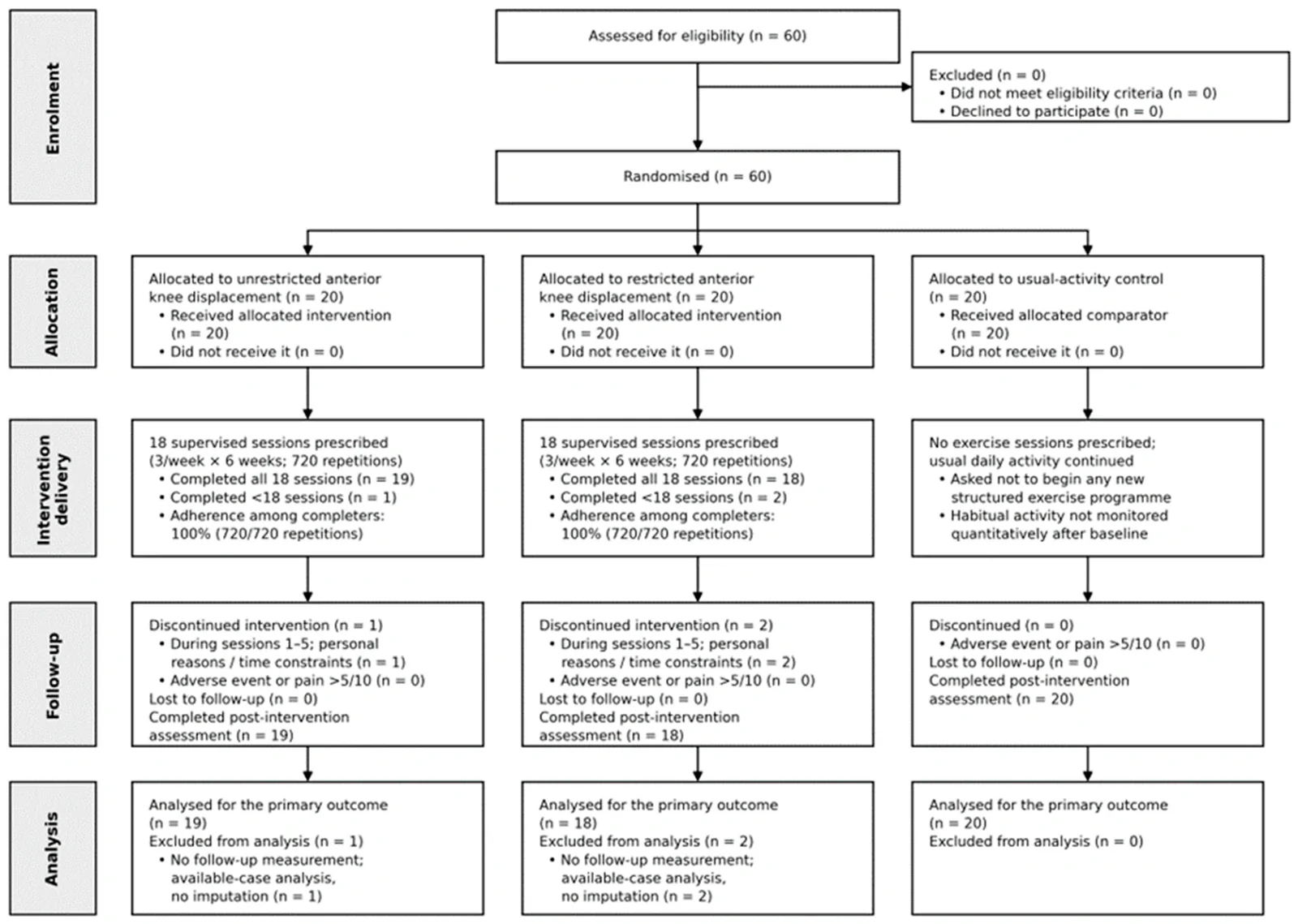
CONSORT 2025 participant-flow diagram. Sixty participants were randomised 1:1:1 by an independent researcher from a pre-generated, randomly permuted allocation list of fixed composition (20 entries per group). Both exercise arms were prescribed 18 physiotherapist-supervised bodyweight squat sessions delivered by synchronous video call three times per week for six weeks (cumulative 720 repetitions). Three participants discontinued during sessions 1–5 for personal reasons or time constraints; none discontinued because of an adverse event or exercise-related pain above the protocol-specified 5/10 visual-analogue-scale withdrawal threshold, and no adverse events occurred in any arm. All 37 completers in the two exercise arms attended every prescribed session (100% adherence among completers). The three participants who discontinued contributed no follow-up measurement; their baseline records were not entered into the analysis dataset and no imputation or missing-data sensitivity analysis was performed, so the analysis set of 57 is available-case (complete-case) rather than intention-to-treat. No party was blinded to allocation. This diagram replaces the CONSORT 2010 flow diagram submitted with the original manuscript and reflects the corrected session count of 18.

Sixty participants were assessed for eligibility, and all 60 were randomized, 20 to each group. Three participants discontinued during the first five sessions of the intervention, one in the unrestricted anterior knee displacement group and two in the restricted group, whereas no participant in the control group withdrew. All three cited personal reasons or time constraints; none discontinued because of an adverse event, exercise-related pain, or dissatisfaction with the assigned technique, and none met the protocol-specified withdrawal criterion of exercise-related pain above 5/10. Fifty-seven participants therefore completed the study and were analysed on an available-case basis: 19 in the unrestricted group, 18 in the restricted group, and 20 in the control group (Figure 1). Baseline assessments were completed before randomization for all 60 participants, but the baseline records of the three participants who discontinued were never entered into the analysis dataset and could not be retrieved during revision, and no comparison of their baseline characteristics with those of the completers and no imputation-based sensitivity analysis were performed. Missing follow-up data amounted to 5% of the randomized sample and arose for reasons unrelated to the intervention, so a material influence on the primary conclusion appears unlikely, but this is an expectation rather than a demonstrated result and is listed among the limitations in Section 4.6.

### 3.2. Baseline Characteristics

Baseline demographic and anthropometric characteristics are presented descriptively in Table 1. Table 1 describes the 57 analysed participants rather than the full randomized cohort of 60: the baseline records of the three participants who discontinued were never entered into the electronic study dataset and could not be retrieved during this revision (Section 3.1), so a 20/20/20 baseline table cannot be produced from the source data; this reason is stated explicitly rather than remedied post hoc. Significance tests of baseline characteristics have been removed, because any difference between randomized groups at baseline arises by chance and hypothesis testing of such differences is uninformative. Numerically, age, body mass, height, limb length, sex distribution, and dominant-limb distribution were similar across the three groups, and all 60 participants met the active category of the IPAQ-SF at screening. Baseline values for all outcome variables are given alongside follow-up values in Table 2, Table 3 and Table 4. One baseline imbalance is material to interpretation: left anterior Y Balance Test reach was lower in both exercise groups (unrestricted 62.89 ± 10.46; restricted 61.67 ± 9.31) than in the control group (69.20 ± 7.08), a difference of approximately 7 cm. Because follow-up scores for this outcome were compared without adjustment for baseline, the corresponding between-group comparison is confounded and is not interpreted (Section 2.7).

### 3.3. Primary Outcome: Low-Back-Related Disability

Follow-up Oswestry Disability Index scores did not differ among the three groups, F(2,54) = 1.999, *p* = 0.145, η^2^ = 0.069 (Table 4). Baseline disability was minimal in every group: 5.26 ± 2.68 raw points (10.5%) in the unrestricted group, 6.56 ± 2.81 (13.1%) in the restricted group, and 6.40 ± 2.21 (12.8%) in the control group. Individual baseline scores ranged from 4% to 24%; almost all lay within the minimal-disability band of 0–20%, and the highest observed value of 24% falls just into the moderate-disability band. The previous version stated that all baseline scores lay within the minimal-disability band, which was inconsistent with the reported range and has been corrected. Pairwise differences at follow-up were small: unrestricted versus restricted, −1.2 percentage points (95% CI −4.5 to 2.1); unrestricted versus control, −2.9 (95% CI −5.9 to 0.0); restricted versus control, −1.7 (95% CI −4.6 to 1.2). The upper limit of every interval lies below the commonly cited minimal clinically important difference of approximately 10 percentage points [23,24]. No clinically important between-group difference was therefore observed within the limits of this sample. This wording is deliberately weaker than that used in the previous version: the trial was not designed as an equivalence or non-inferiority study, no margin was prespecified, and the pronounced floor effect described in Section 4.5 complicates interpretation of these intervals, so a clinically meaningful advantage cannot be said to have been excluded. The trial did not meet its primary endpoint.

### 3.4. Secondary Outcomes: Overview

Across the 21 secondary follow-up comparisons presented in Table 2, Table 3 and Table 4, one omnibus test reached statistical significance. Left-hamstring pressure pain threshold differed among groups, F(2,54) = 3.495, *p* = 0.037, η^2^ = 0.115. Games–Howell analysis showed a higher follow-up value in the unrestricted group than in the control group (mean difference 1.084 kgf/cm^2^, 95% CI 0.008 to 2.160; *p* = 0.048). This Games–Howell interval differs from the corresponding entry in Table 5 (1.08, 95% CI 0.19 to 1.97) because the latter is an unadjusted two-group Welch–Satterthwaite interval computed from the group summary statistics, whereas the Games–Howell interval incorporates the adjustment for multiple post hoc comparisons among the three groups; the point estimates are identical and both intervals exclude zero only marginally. The unrestricted and restricted groups did not differ (*p* = 0.983), and the restricted group did not differ from the control group (*p* = 0.093), although the unadjusted mean difference for the latter contrast was of almost the same magnitude as for the former (1.00 versus 1.08 kgf/cm^2^; Table 5), which argues against a technique-specific effect. The remaining 20 secondary comparisons were not statistically significant (*p* = 0.223–0.978), with η^2^ between 0.001 and 0.054. No secondary result met the Bonferroni-adjusted threshold of *p* < 0.0024. Between-group mean differences with 95% confidence intervals for the principal secondary outcomes are given in Table 5, and for all outcomes in Table S1; every interval other than those for left-hamstring pressure pain threshold includes zero.

### 3.5. Pressure Pain Threshold

At follow-up, no pressure-pain-threshold site other than the left hamstring differed among groups (Table 2). Mean post-intervention values at the gluteus medius (10.19–10.35 kgf/cm^2^) and greater trochanter (9.95–10.09 kgf/cm^2^) lay within 10% of the 11 kgf/cm^2^ protocol ceiling, which constrains the scope for detectable increase at these sites and produces right-censored observations (Section 2.6.1). Within-group pre-post comparisons for each site are reported in Table 2 and are descriptive only; they are not interpreted as evidence of differential efficacy, since statistical significance in one group and non-significance in another does not establish a difference between groups. One feature of those within-group results nevertheless bears directly on interpretation: four of the twelve sites changed significantly in the control group under the standardized recalculation described in Section 2.7 (paraspinal, iliac, hamstrings, and knee), which received no intervention, and all four were on the right side. This pattern is discussed in Section 4.2.

### 3.6. Dynamic Balance

At follow-up, no statistically significant differences were observed among groups in any Y Balance Test direction (*p* = 0.223–0.815; Table 3), and every between-group confidence interval included zero (Table S1). Reach directions are reported using the standard anterior, posteromedial, and posterolateral labels defined in Section 2.6.2. The comparison for left anterior reach is confounded by the baseline imbalance described in Section 3.2 and is not interpreted. Within-group changes are reported in Table 3 as descriptive information only; both exercise groups increased in five of six directions, but so did the control group in right anterior and right posterolateral reach, and the between-group analysis provides no evidence that these changes differed by allocation.

### 3.7. Perceived Exertion

Follow-up Modified Borg scores did not differ among groups (*p* = 0.925; Table 4), and the between-group confidence intervals included zero (Table 5). The Modified Borg values reported here are the assessment-level ratings obtained at the two face-to-face visits from all three groups, not the session-level ratings collected only in the exercise groups (Section 2.6.3). Under the standardized recalculation, no within-group Borg change reached statistical significance in any group (control group 2.25 ± 0.91 to 2.10 ± 0.79, *p* = 0.467); the previously reported significant control-group change (*p* < 0.001) was an error carried over from the source thesis and has been removed.

### 3.8. Vertical Jump Flight Time

Follow-up SJ flight time did not differ among groups (F(2,54) = 0.380, *p* = 0.686), nor did follow-up CMJ flight time (F(2,54) = 0.092, *p* = 0.912; Table 4); all between-group confidence intervals were wide and included zero (Table 5). Within groups, SJ flight time increased in the unrestricted group (464.79 ± 63.68 to 505.01 ± 79.59 ms; *p* < 0.001) and the restricted group (479.28 ± 87.08 to 514.84 ± 95.44 ms; *p* < 0.001) but not in the control group (482.53 ± 56.48 to 492.55 ± 60.29 ms; *p* = 0.199), whereas CMJ flight time increased in all three groups, including the control group (519.77 ± 72.36 to 534.34 ± 74.84 ms; *p* = 0.023). These within-group results are descriptive; the absence of between-group differences means they cannot be attributed to the training.

### 3.9. Harms and Unintended Effects

Three participants discontinued the intervention during the first five sessions, all by personal choice: one in the unrestricted group and two in the restricted group (Section 3.1). None of these discontinuations was related to an adverse event. No adverse events, exercise-related injuries, or instances of exercise-related pain exceeding the protocol-specified 5/10 withdrawal threshold occurred in any group during the trial, and no participant met any other protocol-specified withdrawal criterion. The 37 participants who completed the two exercise programmes did so without interruption or modification (Section 2.5).

### 3.10. Post Hoc Baseline-Adjusted Sensitivity Analyses

In response to peer review, analysis of covariance was performed post hoc for the primary outcome and all 21 secondary outcomes, with the follow-up score as the dependent variable, the baseline score as covariate, and randomized group as a fixed factor; full results, adjusted group means with 95% confidence intervals, and Bonferroni-adjusted pairwise comparisons are given in Table S2. The primary result was unchanged: adjusted follow-up Oswestry scores did not differ among groups, F(2,53) = 1.539, *p* = 0.224, partial η^2^ = 0.055. Most secondary conclusions were likewise unchanged. Six of the 21 adjusted secondary comparisons reached *p* < 0.05 uncorrected—left paraspinal, left iliac, and left hamstring pressure pain thresholds, right posteromedial Y Balance Test reach, and SJ and CMJ flight times—and in every case the adjusted differences favoured the exercise groups over the control group. Two fell below the Bonferroni reference threshold of *p* < 0.0024: left hamstring pressure pain threshold, F(2,53) = 7.234, *p* = 0.002, partial η^2^ = 0.214, consistent with the unadjusted finding in Section 3.4, and right posteromedial reach, F(2,53) = 7.554, *p* = 0.001, partial η^2^ = 0.222, with adjusted exercise-versus-control differences of approximately 4.5 cm. The homogeneity-of-regression-slopes assumption held for all outcomes except right knee pressure pain threshold (group × baseline interaction *p* = 0.044); the ANCOVA estimate for that outcome is therefore considered unreliable and no inference is drawn from it. Because these analyses were not prespecified and the trial remains exploratory, these results are reported as sensitivity findings and are hypothesis-generating; they do not alter the primary conclusion.

## 4. Discussion

### 4.1. Principal Findings

This trial compared unrestricted anterior knee displacement, restricted anterior knee displacement, and usual activity. The primary outcome was negative: low-back-related disability did not differ among groups at follow-up, and the confidence interval for every pairwise difference lay below the commonly cited minimal clinically important difference for the Oswestry Disability Index. No clinically important between-group difference was therefore observed within the limits of this sample. That statement is deliberately bounded. The trial was not designed as an equivalence or non-inferiority study, no margin was prespecified, the sample was powered only for large effects (η^2^ ≈ 0.15), and the instrument was applied in a population with almost no disability to lose, so the confidence intervals cannot be read as excluding a clinically meaningful advantage for either pattern. The secondary results were likewise predominantly null: one of 21 comparisons reached *p* < 0.05, none met the multiplicity-adjusted threshold, and every between-group confidence interval other than that for left-hamstring pressure pain threshold included zero. Small and moderate differences in pressure pain sensitivity, dynamic balance, and jump performance remain entirely compatible with these data.

### 4.2. The Isolated Left-Hamstring Finding and the Role of the Control Group

The only significant omnibus finding in the unadjusted analysis was a secondary outcome, left-hamstring pressure pain threshold, with a pairwise difference between the unrestricted and control groups. Several features warrant caution. It was one of 21 secondary outcomes evaluated without multiplicity correction, and *p* = 0.037 does not approach the Bonferroni-adjusted threshold of *p* < 0.0024. In the baseline-adjusted sensitivity analysis this contrast strengthened rather than disappeared (adjusted unrestricted-versus-control difference 1.14 kgf/cm^2^, 95% CI 0.39 to 1.89; omnibus *p* = 0.002, below the reference threshold), so it is not an artifact of the unadjusted analysis, but it remains exploratory. The lower confidence limit for the pairwise difference was 0.008 on the Games–Howell interval, effectively touching zero. The finding was unilateral, whereas the right hamstring showed almost no between-group separation (F(2,54) = 0.080). The restricted group also exceeded the control group by a nearly identical unadjusted margin (1.00 versus 1.08 kgf/cm^2^) without reaching significance, which is difficult to reconcile with an effect specific to unrestricted knee travel. And the difference was driven as much by the control group as by the intervention: over the same interval the control group gained 0.94 kgf/cm^2^ at the right hamstring (*p* = 0.001, d = 0.63) but lost 0.13 kgf/cm^2^ at the left, an asymmetry within an untreated group that is difficult to attribute to any physiological process and is more consistent with measurement variability. The result is best regarded as hypothesis-generating and is given correspondingly little weight in the Abstract and Conclusions.

The behaviour of the control group is informative more generally. Viewed without a comparator, the within-group findings appear favourable: both exercise groups improved at several algometry sites, in five Y Balance Test directions, and in jump flight time. However, the control group also changed at four algometry sites, in right anterior and right posterolateral reach, and in CMJ flight time. This pattern does not demonstrate that the exercise-group changes were measurement artifacts, but it is compatible with familiarization, ordinary test–retest variation, rater or procedural drift, regression to the mean, and changes in habitual activity that were not monitored after baseline. At the same time, the post hoc baseline-adjusted sensitivity analyses strengthened rather than weakened several exercise-versus-control contrasts—most consistently left-sided pressure pain thresholds, right posteromedial reach, and jump flight times (Section 3.10, Table S2)—which tempers a purely artifactual reading of the within-group changes; because these analyses were not prespecified, they remain hypothesis-generating. Pressure algometry has acceptable reliability when procedures are standardized, yet repeated values remain sensitive to assessor technique and participant response [18], and the present measurements were made by a single unblinded rater. Y Balance Test performance can improve with repeated attempts before reaching a plateau [29], and systematic-review evidence indicates that measurement error must be considered when interpreting small reach-distance changes [20]. Repeated jump testing can also be influenced by procedural and time-of-day effects [30]. These considerations are the reason the within-group analyses are reported descriptively and the between-group comparisons are given priority.

### 4.3. Training Stimulus and the Intended Technique Contrast

The modest training stimulus offers a plausible explanation for the lack of clear between-group effects. The program progressed from one set of 20 to two sets of 30 controlled bodyweight repetitions, without external resistance, maximal-velocity intent, or a prescribed proximity to muscular failure. Low-load, high-repetition exercise may improve local endurance, but meaningful strength or power adaptation depends on sufficient effort, progression, and task specificity [31]. Vertical-jump gains are more consistently observed after structured plyometric or resistance programs that emphasize rapid force production and progressive overload [32]. Because participants were already physically active, the incremental overload relative to habitual activity may have been especially small. No outcome directly indexed the trained task itself—neither achieved squat depth during training nor knee-extensor strength was measured—so the absence of adaptation is inferred rather than demonstrated.

A more fundamental constraint is that anterior knee displacement was not isolated from squat depth. The unrestricted group descended to a self-selected comfortable depth, whereas the restricted group targeted approximately 90° of knee flexion. Any difference between groups would therefore have reflected a combined contrast in knee trajectory, attainable depth, trunk inclination, hip contribution, and probably total mechanical work. Range of motion can influence knee-joint exposure, muscle development, and training adaptation [33,34,35,36], while squat variations under progressive loading may yield selective strength differences with broadly comparable hypertrophic adaptation [37]. Compounding this, no manipulation check confirmed the anterior knee travel or depth actually achieved during the training sessions, and ankle dorsiflexion range of motion—an anatomical determinant of both—was not measured, so the groups cannot be shown to have been comparable in their capacity to execute the assigned pattern. The comparison delivered is therefore between two squat-instruction packages, not between isolated restricted and unrestricted anterior knee displacement, and the findings should be read strictly in those terms.

### 4.4. Dynamic Balance and Jump Performance: Task Specificity

The absence of a between-group Y Balance Test effect is consistent with task specificity. Resistance exercise can improve some balance outcomes, but pooled effects for the Y Balance Test are not consistently significant, and gains in general strength or power do not necessarily transfer to an untrained multidirectional reach task [38,39]. The present intervention trained bilateral squatting rather than unilateral stance, multidirectional reaching, or perturbation recovery. The control-group increase in right anterior reach and documented learning effects further suggest that repeated testing contributed to the observed pre-post pattern [20,29]. The jump results reflect the same principle. SJ and CMJ performance are influenced by rapid force production, stretch-shortening-cycle behaviour, coordination, and testing procedures, whereas the intervention emphasized slow, metronome-paced squatting rather than maximal or ballistic intent. Longer-term jump gains are more consistently produced by plyometric or progressively loaded training [32], and visual feedback during jump-squat training can enhance selected sport-performance outcomes [40]. CMJ flight time increased in all three groups and SJ flight time increased only in the exercise groups, but the absence of between-group differences prevents these patterns from being interpreted as differential training effects, and repeated-testing effects provide an additional plausible explanation [30].

### 4.5. Choice of Primary Outcome and Practical Implications

The null primary result cannot be separated from the properties of the instrument in this sample, and the mismatch is one of design rather than merely of interpretation. The Oswestry Disability Index was developed to quantify disability in adults with low back pain, and its responsiveness depends on there being disability to reduce. Every participant here was healthy, physically active, and asymptomatic by eligibility criteria: baseline scores occupied only the lowest quarter of the scale (4% to 24%), leaving little room for improvement and none for a clinically meaningful one. Selecting this endpoint as the primary outcome for this population was, in retrospect, an error of design; a performance or biomechanical endpoint would have been substantially more informative, and the sample size should have been derived from it. The statistically significant 2.2-percentage-point reduction in the restricted group is roughly one fifth of the minimal clinically important difference and lies within the measurement error of the instrument. The IKDC scores raised a similar concern and have been withdrawn because their scoring could not be verified against the original source records (Section 2.6.3). The Oswestry outcome provides reassurance that neither squat pattern produced symptom burden over six weeks, but it was never likely to discriminate between training conditions in an asymptomatic sample.

The practical implication is correspondingly narrow. These data do not show that knee position is irrelevant, nor do they license a recommendation that squat technique should be individualized rather than governed by a general rule; a single null trial in asymptomatic young adults performing unloaded squats cannot support a prescriptive claim in either direction, and the previous version overstated this. What can be said is that, within the specific conditions studied, restricting anterior knee displacement conferred no detectable advantage for pressure sensitivity, dynamic balance, or jump flight time, and permitting natural anterior knee travel produced no consistent superiority; neither pattern was associated with any adverse event or with exercise-related pain exceeding the withdrawal threshold. Clinicians choosing between these patterns must continue to weigh anthropometry, ankle and hip mobility, symptoms, desired depth, external load, and the intended adaptation on the basis of the acute biomechanical literature, which remains the primary evidence base for this decision. Strengths of the trial include the usual-activity control group, whose behaviour proved essential to interpreting the within-group changes; a repeated supervised intervention rather than an acute laboratory comparison; complete adherence among participants who completed the protocol; concealed allocation from a list prepared and held outside the research team; and an outcome battery spanning symptom-related and functional domains.

### 4.6. Limitations and Future Research

Several limitations qualify every statement above. The primary outcome was mismatched to the population and subject to a pronounced floor effect. The sample size was calculated from functional-performance literature rather than from a disability-specific effect estimate, and the assumed effect (0.799) was large; the trial could detect only large between-group differences (η^2^ ≈ 0.15) and cannot exclude small or moderate ones, demonstrate equivalence, or support non-inferiority. Most importantly for the analysis, the primary between-group comparisons were made on follow-up values without adjustment for baseline; post hoc ANCOVA sensitivity analyses have now been added (Section 3.10, Table S2), but they were not prespecified and the primary presentation remains unadjusted. For left anterior Y Balance Test reach, the one outcome with a baseline imbalance, the unadjusted comparison remains uninterpreted; its baseline-adjusted group effect did not reach the conventional significance level (F(2,53) = 3.142, *p* = 0.051). No party was blinded, and pressure algometry in particular was performed by a single unblinded rater in a fixed site order, with an unquantified number of observations at the 11 kgf/cm^2^ protocol ceiling producing censored data that violate the assumptions of the parametric tests applied. The analysis was available-case rather than intention-to-treat: three of 60 randomized participants contributed no follow-up data, their baseline records were not entered into the analysis dataset, and no sensitivity analysis for missing data was performed. Twenty-one secondary outcomes were examined without multiplicity correction, so approximately one false-positive result would be expected by chance alone, and several reported outcomes were not entered in the trial registry (Section 2.1). Anterior knee displacement was not isolated from squat depth, no kinematic manipulation check was performed, the instructional depth measurements were not retained, and ankle dorsiflexion was not measured. Habitual activity in the control group was not monitored after baseline, and IPAQ-SF scores were not retained for analysis. Inter-rater reliability for the Y Balance Test was not quantified despite the use of two raters. The IKDC outcome was withdrawn during revision because its scoring could not be verified against the original source records; this withdrawal narrows the self-reported outcome battery and reflects a weakness of the trial’s data management, as does the absence of retained session-level attendance records (Section 2.5). Outcomes were assessed only immediately after the intervention, so persistence is unknown. The findings apply to healthy, physically active adults aged 18–30 years performing short-term, low-intensity, unloaded bodyweight squats and should not be extrapolated to symptomatic knee or low-back populations, postoperative rehabilitation, older or sedentary adults, heavy barbell or high-intensity strength training, or injury prevention.

Future trials should select a primary outcome with adequate responsiveness in the population studied and derive the sample size from it; in asymptomatic active adults, a performance or biomechanical endpoint is likely to be far more informative than a disability index. Trials should match squat depth and total mechanical work between conditions, screen and report ankle dorsiflexion range, and verify the intended kinematic contrast with an objective manipulation check rather than relying on instruction alone. Outcome assessment should be blinded where the outcome permits, algometry standardized with a documented application rate and no ceiling effect, and repeated measures analysed with baseline-adjusted analysis of covariance or a group-by-time mixed model, reported as adjusted between-group estimates with 95% confidence intervals. A progressively overloaded stimulus of sufficient duration is needed before any conclusion about adaptation can be drawn. Combining three-dimensional kinematics, joint moments, electromyography, strength testing, jump force-time analysis, quantified adherence, and longer-term follow-up would clarify whether restricted and unrestricted squat strategies create distinct biomechanical adaptations even when gross functional outcomes appear similar.

## 5. Conclusions

A progressive 18-session bodyweight squat program performed with either unrestricted or restricted anterior knee displacement did not improve low-back-related disability, the registered primary outcome, relative to the alternative squat pattern or to usual activity, and no consistent advantage emerged across the 21 secondary outcomes. Post hoc baseline-adjusted sensitivity analyses left the primary conclusion unchanged while strengthening several exploratory exercise-versus-control contrasts (Table S2). Comparable within-group changes in the untreated control group indicate that the observed pre-post improvements cannot be attributed confidently to training. In the unadjusted analysis, the left-hamstring pressure-pain-threshold difference was the only secondary contrast to reach *p* < 0.05 and did not survive correction for multiplicity; in the post hoc baseline-adjusted sensitivity analyses, this contrast and right posteromedial reach fell below the multiplicity-adjusted reference threshold, and six adjusted contrasts reached uncorrected *p* < 0.05 (Section 3.10)—all post hoc and exploratory. The unadjusted left-hamstring finding was of almost identical magnitude in the restricted group, and was driven in part by an unexplained side-to-side asymmetry in the control group; it should be treated as hypothesis-generating. Within the specific conditions of this short-term bodyweight-squat intervention in healthy, physically active young adults aged 18–30 years, restricting anterior knee displacement did not provide a detectable advantage. No clinically important between-group difference was observed within the limits of this sample. Because the primary outcome was subject to a pronounced floor effect, the trial was powered only for large effects, the between-group analyses were not adjusted for baseline, and no equivalence margin was prespecified, these results do not demonstrate that the two techniques are biomechanically equivalent or equally safe under other training and rehabilitation conditions, and they do not support any general prescriptive rule about knee position during squatting.

## Figures and Tables

**Table 1:** Baseline demographic and anthropometric characteristics.

Characteristic	Unrestricted (*n* = 19)	Restricted (*n* = 18)	Control (*n* = 20)
Age, years	26.21 ± 2.55	26.72 ± 2.37	25.55 ± 2.14
Body mass, kg	73.32 ± 14.38	73.06 ± 13.87	72.35 ± 16.82
Height, cm	175.89 ± 10.17	174.11 ± 9.48	173.40 ± 9.54
Limb length, cm	85.47 ± 5.08	85.17 ± 5.96	83.90 ± 5.46
Sex, male/female	12/7	11/7	11/9
Dominant limb, right/left	16/3	16/2	16/4

Data are mean ± standard deviation or count. Baseline characteristics are presented descriptively; significance tests of baseline differences between randomized groups have been removed because such tests are uninformative after randomization. All participants met the active category of the IPAQ-SF at screening; numerical MET min/week scores were not retained for analysis. Group counts agree with the CONSORT participant flow diagram and with the degrees of freedom reported throughout (F(2,54)).

**Table 2:** Pressure-algometry values before and after the intervention.

Outcome	Group	Baseline Mean ± SD	Post Mean ± SD	Within-Group *p*	d	Follow-Up F/*p*/η^2^
L1–L3, right	Unrestricted	8.84 ± 1.85	9.30 ± 1.51	0.095	0.27	0.432/0.652/0.016
	Restricted	8.54 ± 1.65	9.18 ± 1.65	0.041 *	0.39	
	Control	8.86 ± 1.55	9.61 ± 1.30	<0.001 *†	0.53	
L1–L3, left	Unrestricted	8.94 ± 1.76	9.60 ± 1.23	0.039 *	0.44	1.456/0.242/0.051
	Restricted	8.53 ± 1.84	9.36 ± 1.68	0.111 †	0.47	
	Control	9.03 ± 1.44	8.84 ± 1.38	0.106 †	0.13	
Iliac region, right	Unrestricted	8.71 ± 1.73	9.14 ± 1.68	0.027 *	0.25	0.177/0.838/0.007
	Restricted	7.80 ± 1.88	8.86 ± 1.62	0.005 *†	0.61	
	Control	8.69 ± 1.00	8.97 ± 1.04	0.018 *†	0.28	
Iliac region, left	Unrestricted	8.76 ± 1.94	9.01 ± 1.77	0.151	0.13	1.201/0.309/0.043
	Restricted	8.01 ± 2.05	9.31 ± 1.36	0.002 *	0.76	
	Control	8.53 ± 1.47	8.54 ± 1.47	0.669 †	0.01	
Gluteus medius, right	Unrestricted	10.39 ± 1.05	10.25 ± 1.03	0.733 †	0.14	0.379/0.687/0.014
	Restricted	10.39 ± 1.24	10.51 ± 0.93	0.679 †	0.11	
	Control	10.16 ± 1.05	10.28 ± 1.07	0.309 †	0.11	
Gluteus medius, left	Unrestricted	10.21 ± 1.39	10.18 ± 1.12	0.765 †	0.02	0.752/0.476/0.027
	Restricted	10.14 ± 1.34	10.43 ± 1.23	0.413 †	0.23	
	Control	10.06 ± 1.16	9.96 ± 1.17	0.516	0.09	
Hamstrings, right	Unrestricted	8.97 ± 1.82	9.30 ± 1.35	0.138	0.21	0.080/0.923/0.003
	Restricted	9.06 ± 1.78	9.49 ± 1.40	0.046 *	0.27	
	Control	8.41 ± 1.43	9.35 ± 1.56	0.001 *†	0.63	
Hamstrings, left	Unrestricted	8.63 ± 2.08	9.68 ± 1.45	0.002 *	0.60	3.495/0.037 */0.115
	Restricted	9.17 ± 1.66	9.60 ± 1.53	0.143	0.27	
	Control	8.73 ± 1.54	8.60 ± 1.29	0.611	0.09	
Knee, right	Unrestricted	7.40 ± 1.88	8.01 ± 1.73	0.011 *	0.34	0.633/0.535/0.023
	Restricted	8.06 ± 2.09	8.54 ± 1.57	0.195	0.27	
	Control	8.01 ± 1.48	8.48 ± 1.45	0.001 *†	0.32	
Knee, left	Unrestricted	7.66 ± 1.85	8.45 ± 1.53	0.002 *†	0.47	0.022/0.978/0.001
	Restricted	8.32 ± 1.96	8.46 ± 1.79	0.733	0.07	
	Control	8.31 ± 1.20	8.36 ± 1.36	0.867 †	0.04	
Greater trochanter, right	Unrestricted	9.92 ± 1.61	10.22 ± 1.28	0.018 *†	0.21	0.200/0.819/0.007
	Restricted	9.64 ± 1.73	10.09 ± 1.53	0.058	0.28	
	Control	9.59 ± 1.39	9.95 ± 1.25	0.053	0.27	
Greater trochanter, left	Unrestricted	9.86 ± 1.45	10.12 ± 1.31	0.131	0.19	0.996/0.376/0.036
	Restricted	9.99 ± 1.62	10.14 ± 1.37	0.365 †	0.10	
	Control	9.49 ± 1.32	9.60 ± 1.37	0.342	0.08	

Values are mean ± SD in kgf/cm^2^; all sites were secondary outcomes. Within-group *p*: paired *t* test, or Wilcoxon signed-rank test (†) where the Shapiro–Wilk test indicated non-normal differences; descriptive only (Section 2.7). d, Cohen’s d. Follow-up columns: F(2,54), *p*, and η^2^ from one-way ANOVA, unadjusted for baseline. * *p* < 0.05, uncorrected (Bonferroni reference *p* < 0.0024). Values at the 11 kgf/cm^2^ ceiling are right-censored (Section 2.6.1).

**Table 3:** Y Balance Test reach distance (cm) before and after the intervention.

Outcome	Group	Baseline Mean ± SD	Post Mean ± SD	Within-Group *p*	d	Follow-Up F/*p*/η^2^
Right anterior	Unrestricted	62.95 ± 10.96	65.89 ± 11.60	0.005 *	0.26	1.541/0.223/0.054
	Restricted	62.44 ± 10.13	66.94 ± 9.53	<0.001 *†	0.46	
	Control	67.35 ± 6.66	70.90 ± 6.53	<0.001 *†	0.54	
Left anterior	Unrestricted	62.89 ± 10.46	65.89 ± 11.42	0.005 *†	0.27	0.588/0.559/0.021
	Restricted	61.67 ± 9.31	65.72 ± 9.25	0.040 *†	0.44	
	Control	69.20 ± 7.08	68.60 ± 6.70	0.365	0.09	
Right posterolateral	Unrestricted	65.42 ± 13.79	69.79 ± 12.45	0.003 *	0.33	0.248/0.781/0.009
	Restricted	67.89 ± 16.98	71.72 ± 14.62	0.003 *†	0.24	
	Control	71.00 ± 7.43	72.30 ± 6.59	0.017 *†	0.19	
Right posteromedial	Unrestricted	68.95 ± 13.07	72.63 ± 13.35	<0.001 *	0.28	1.424/0.250/0.050
	Restricted	74.00 ± 15.12	77.22 ± 13.59	0.024 *	0.22	
	Control	71.95 ± 8.23	70.85 ± 8.31	0.189	0.13	
Left posterolateral	Unrestricted	67.11 ± 15.38	71.37 ± 13.59	0.007 *†	0.29	0.357/0.701/0.013
	Restricted	68.83 ± 15.07	73.39 ± 13.07	0.001 *	0.32	
	Control	73.30 ± 8.07	74.45 ± 7.05	0.339 †	0.15	
Left posteromedial	Unrestricted	72.79 ± 14.97	73.84 ± 14.66	0.174	0.07	0.205/0.815/0.008
	Restricted	70.33 ± 14.63	71.33 ± 14.26	0.355	0.07	
	Control	72.30 ± 7.29	72.25 ± 5.45	0.948	0.01	

Values are unnormalized reach distance in centimetres, recorded to the nearest centimetre (Section 2.6.2); all directions were secondary outcomes, labelled by the standard anterior/posteromedial/posterolateral convention. Within-group *p* and d as in Table 2. † indicates a Wilcoxon signed-rank test (Section 2.7). Follow-up columns: F(2,54), *p*, and η^2^ from one-way ANOVA. * *p* < 0.05, uncorrected. Left anterior reach is confounded by baseline imbalance and is not interpreted (Section 3.6).

**Table 4:** Perceived exertion and vertical jump flight time.

Outcome	Group	Baseline Mean ± SD	Post Mean ± SD	Within-Group *p*	d	Follow-Up F/*p*/η^2^
Modified Borg	Unrestricted	2.05 ± 1.03	2.05 ± 0.71	1.000	0.00	0.078/0.925/0.003
	Restricted	2.50 ± 1.30	2.00 ± 0.84	0.146 †	0.47	
	Control	2.25 ± 0.91	2.10 ± 0.79	0.467 †	0.18	
Oswestry score	Unrestricted	5.26 ± 2.68	4.84 ± 2.52	0.429	0.16	1.999/0.145/0.069
	Restricted	6.56 ± 2.81	5.44 ± 2.36	0.039 *†	0.43	
	Control	6.40 ± 2.21	6.30 ± 1.98	0.834	0.05	
SJ flight time, ms	Unrestricted	464.79 ± 63.68	505.01 ± 79.59	<0.001 *	0.56	0.380/0.686/0.014
	Restricted	479.28 ± 87.08	514.84 ± 95.44	<0.001 *	0.39	
	Control	482.53 ± 56.48	492.55 ± 60.29	0.199	0.17	
CMJ flight time, ms	Unrestricted	493.68 ± 65.72	535.19 ± 78.40	<0.001 *	0.58	0.092/0.912/0.003
	Restricted	497.22 ± 68.17	544.28 ± 81.71	<0.001 *	0.63	
	Control	519.77 ± 72.36	534.34 ± 74.84	0.023 *	0.20	

Values are mean ± SD. The Oswestry Disability Index is the outcome designated as primary; all other measures are secondary (Section 2.1). Oswestry values are raw totals (0–50); percentages are twice these values. Within-group *p* and d as in Table 2. † indicates a Wilcoxon signed-rank test (Section 2.7). Follow-up columns: F(2,54), *p*, and η^2^ from one-way ANOVA, unadjusted for baseline. * *p* < 0.05, uncorrected. SJ, squat jump; CMJ, countermovement jump.

**Table 5:** Between-group differences in follow-up scores for the primary outcome and the principal secondary outcomes.

Outcome	Unrestricted vs. Restricted	Unrestricted vs. Control	Restricted vs. Control
ODI, percentage points	−1.20 (−4.46 to 2.06)	−2.92 (−5.88 to 0.04)	−1.72 (−4.62 to 1.18)
PPT left hamstring, kgf/cm^2^	0.08 (−0.92 to 1.08)	1.08 (0.19 to 1.97)	1.00 (0.06 to 1.94)
PPT right hamstring, kgf/cm^2^	−0.19 (−1.11 to 0.73)	−0.05 (−1.00 to 0.90)	0.14 (−0.83 to 1.11)
PPT L1–L3 left, kgf/cm^2^	0.24 (−0.75 to 1.23)	0.76 (−0.09 to 1.61)	0.52 (−0.50 to 1.54)
PPT L1–L3 right, kgf/cm^2^	0.12 (−0.94 to 1.18)	−0.31 (−1.23 to 0.61)	−0.43 (−1.42 to 0.56)
YBT right anterior, cm	−1.05 (−8.12 to 6.02)	−5.01 (−11.23 to 1.21)	−3.96 (−9.43 to 1.51)
YBT right posteromedial, cm	−4.59 (−13.59 to 4.41)	1.78 (−5.54 to 9.10)	6.37 (−1.22 to 13.96)
YBT right posterolateral, cm	−1.93 (−11.03 to 7.17)	−2.51 (−9.10 to 4.08)	−0.58 (−8.33 to 7.17)
Modified Borg, points	0.05 (−0.47 to 0.57)	−0.05 (−0.54 to 0.44)	−0.10 (−0.64 to 0.44)
SJ flight time, ms	−9.83 (−68.76 to 49.10)	12.46 (−33.69 to 58.61)	22.29 (−31.42 to 76.00)
CMJ flight time, ms	−9.09 (−62.61 to 44.43)	0.85 (−48.94 to 50.64)	9.94 (−41.87 to 61.75)

Values are unadjusted between-group mean differences in follow-up scores with 95% confidence intervals, calculated from the group means, standard deviations, and sample sizes reported in Table 2, Table 3 and Table 4 using the Welch–Satterthwaite approximation. Positive values favour the first-named group. These estimates are not adjusted for baseline scores; the limitations of this are set out in Section 2.7 and Section 4.6. Corresponding estimates for all 21 secondary outcomes are given in Table S1. ODI, Oswestry Disability Index; PPT, pressure pain threshold; YBT, Y Balance Test; SJ, squat jump; CMJ, countermovement jump.

## Data Availability

The anonymized participant-level dataset generated and analysed during the current study is submitted with this revision as Supplementary Data S1 (including the baseline demographic variables underlying Table 1), together with the analysis code that reproduces every statistic in Table 2, Table 3, Table 4 and Table 5, Tables S1 and S2 (Supplementary Code S1), and remains additionally available from the corresponding author. The registry’s individual-participant-data sharing field was revised in the August 2026 amendment so that it is consistent with this declaration; that amendment was awaiting posting when this manuscript was submitted (Section 2.1).

## Supplementary Materials

The following supporting information can be downloaded at: https://www.mdpi.com/article/10.3390/jcm15187162/s1, Table S1. Unadjusted follow-up comparisons for all 21 secondary outcomes with 95% confidence intervals; Table S2. Post hoc baseline-adjusted (ANCOVA) sensitivity analyses for the primary and all secondary outcomes; Supplementary File S1. The original February 2025 ClinicalTrials.gov registration record (archived version); Supplementary File S2. An item-by-item verification report of all reported statistics against the dataset; and the pre-computed output log of the analysis code, so that every statistic can be checked without running any software; Supplementary Data S1. The anonymized participant-level dataset; Supplementary Code S1. The analysis code reproducing all reported statistics; Supplementary File S3. The completed CONSORT 2025 checklist [12,13].
