# Supplementary material for: Unrestricted Versus Restricted Anterior Knee Displacement During Bodyweight Squat Training in Physically Active Young Adults: An Exploratory Three-Arm Randomized Controlled Trial
Source: J Clin Med. 2026 Sep 15;15(18):7162. doi: 10.3390/jcm15187162 (PMC13607129; doi:10.3390/jcm15187162)
Supplement: Supplementary file 1 [file jcm-15-07162-s001.zip › 01_Table_S1.pdf]

Table S1. Unadjusted follow-up comparisons for all 21 secondary outcomes: group means, one-way ANOVA, and pairwise mean differences with 95% confidence intervals.

Outcome	Unrestricted, follow-up mean ± SD (n = 19)	Restricted, follow-up mean ± SD (n = 18)	Control, follow-up mean ± SD (n = 20)	F(2,54)	p	η²	Unrestricted – Restricted, mean diff (95% CI)	Unrestricted – Control, mean diff (95% CI)	Restricted – Control, mean diff (95% CI)
Oswestry Disability Index, %	4.84 ± 2.52	5.44 ± 2.36	6.30 ± 1.98	1.999	.145	.069	-0.60 (-2.11 to 0.91)	-1.46 (-2.93 to 0.01)	-0.86 (-2.35 to 0.64)
PPT paraspinal (L1–L3), right, kgf/cm²	9.30 ± 1.51	9.18 ± 1.65	9.61 ± 1.30	0.432	.652	.016	0.12 (-0.86 to 1.10)	-0.31 (-1.26 to 0.65)	-0.43 (-1.40 to 0.54)
PPT paraspinal (L1–L3), left, kgf/cm²	9.61 ± 1.23	9.36 ± 1.68	8.84 ± 1.38	1.456	.242	.051	0.24 (-0.70 to 1.19)	0.77 (-0.16 to 1.69)	0.52 (-0.41 to 1.46)
PPT iliac, right, kgf/cm²	9.14 ± 1.68	8.86 ± 1.62	8.97 ± 1.04	0.177	.838	.007	0.28 (-0.68 to 1.25)	0.17 (-0.77 to 1.11)	-0.11 (-1.07 to 0.84)
PPT iliac, left, kgf/cm²	9.01 ± 1.77	9.31 ± 1.36	8.54 ± 1.47	1.201	.309	.043	-0.29 (-1.31 to 0.73)	0.48 (-0.52 to 1.47)	0.77 (-0.24 to 1.78)
PPT gluteus medius, right, kgf/cm²	10.25 ± 1.03	10.51 ± 0.93	10.28 ± 1.07	0.379	.687	.014	-0.26 (-0.93 to 0.41)	-0.03 (-0.68 to 0.63)	0.24 (-0.42 to 0.90)
PPT gluteus medius, left, kgf/cm²	10.18 ± 1.12	10.43 ± 1.23	9.96 ± 1.17	0.752	.476	.027	-0.25 (-1.02 to 0.53)	0.22 (-0.53 to 0.98)	0.47 (-0.30 to 1.23)
PPT hamstrings, right, kgf/cm²	9.30 ± 1.35	9.49 ± 1.40	9.35 ± 1.56	0.080	.923	.003	-0.18 (-1.13 to 0.77)	-0.05 (-0.97 to 0.88)	0.14 (-0.80 to 1.07)
PPT hamstrings, left, kgf/cm²	9.68 ± 1.45	9.60 ± 1.53	8.60 ± 1.29	3.495	.037	.115	0.09 (-0.85 to 1.03)	1.08 (0.17 to 2.00)	1.00 (0.07 to 1.92)
PPT knee, right, kgf/cm²	8.01 ± 1.73	8.54 ± 1.57	8.49 ± 1.45	0.633	.535	.023	-0.53 (-1.58 to 0.52)	-0.47 (-1.49 to 0.55)	0.05 (-0.98 to 1.09)
PPT knee, left, kgf/cm²	8.45 ± 1.53	8.46 ± 1.79	8.36 ± 1.36	0.022	.978	.001	-0.01 (-1.04 to 1.02)	0.08 (-0.92 to 1.09)	0.10 (-0.92 to 1.11)
PPT greater trochanter, right, kgf/cm²	10.22 ± 1.28	10.10 ± 1.53	9.95 ± 1.24	0.200	.819	.007	0.13 (-0.77 to 1.02)	0.27 (-0.59 to 1.14)	0.15 (-0.73 to 1.03)
PPT greater trochanter, left, kgf/cm²	10.12 ± 1.31	10.14 ± 1.37	9.60 ± 1.37	0.996	.376	.036	-0.02 (-0.91 to 0.87)	0.52 (-0.35 to 1.39)	0.54 (-0.34 to 1.42)
YBT right anterior, cm	65.89 ± 11.59	66.94 ± 9.53	70.90 ± 6.53	1.541	.223	.054	-1.05 (-7.25 to 5.15)	-5.01 (-11.04 to 1.03)	-3.96 (-10.08 to 2.17)
YBT left anterior, cm	65.89 ± 11.42	65.72 ± 9.25	68.60 ± 6.70	0.588	.559	.021	0.17 (-5.95 to 6.29)	-2.71 (-8.67 to 3.26)	-2.88 (-8.92 to 3.17)
YBT right posterolateral, cm	69.79 ± 12.45	71.72 ± 14.62	72.30 ± 6.59	0.248	.781	.009	-1.93 (-9.57 to 5.71)	-2.51 (-9.95 to 4.93)	-0.58 (-8.12 to 6.97)
YBT left posterolateral, cm	71.37 ± 13.59	73.39 ± 13.07	74.45 ± 7.04	0.357	.701	.013	-2.02 (-9.62 to 5.58)	-3.08 (-10.48 to 4.32)	-1.06 (-8.57 to 6.45)
YBT right posteromedial, cm	72.63 ± 13.35	77.22 ± 13.59	70.85 ± 8.31	1.424	.250	.050	-4.59 (-12.44 to 3.26)	1.78 (-5.87 to 9.43)	6.37 (-1.39 to 14.13)
YBT left posteromedial, cm	73.84 ± 14.66	71.33 ± 14.26	72.25 ± 5.45	0.205	.815	.008	2.51 (-5.46 to 10.48)	1.59 (-6.17 to 9.36)	-0.92 (-8.79 to 6.96)
Modified Borg (assessment-level), 0–10	2.05 ± 0.71	2.00 ± 0.84	2.10 ± 0.79	0.078	.925	.003	0.05 (-0.46 to 0.57)	-0.05 (-0.55 to 0.45)	-0.10 (-0.61 to 0.41)
SJ flight time, ms	505.01 ± 79.59	514.84 ± 95.44	492.55 ± 60.29	0.380	.686	.014	-9.83 (-62.00 to 42.34)	12.46 (-38.35 to 63.27)	22.29 (-29.24 to 73.82)
CMJ flight time, ms	535.20 ± 78.40	544.28 ± 81.71	534.35 ± 74.84	0.092	.912	.003	-9.09 (-60.68 to 42.51)	0.85 (-49.40 to 51.10)	9.94 (-41.02 to 60.90)

Values as observed at follow-up (week 6); analyses on the available-case set (n = 57). F, p, and η² from one-way ANOVA on follow-up scores, unadjusted for baseline (Section 2.7). Pairwise mean differences with t-based 95% CIs, uncorrected. All secondary outcomes are exploratory; the Bonferroni reference threshold is p < .0024 (.05/21). PPT, pressure pain threshold; YBT, Y Balance Test (unnormalized reach distance, cm); SJ, squat jump; CMJ, countermovement jump. The Oswestry row (primary outcome) is included for completeness and duplicates the values reported in the main text.
