# Supplementary material for: Unrestricted Versus Restricted Anterior Knee Displacement During Bodyweight Squat Training in Physically Active Young Adults: An Exploratory Three-Arm Randomized Controlled Trial
Source: J Clin Med. 2026 Sep 15;15(18):7162. doi: 10.3390/jcm15187162 (PMC13607129; doi:10.3390/jcm15187162)
Supplement: Supplementary file 1 [file jcm-15-07162-s001.zip › 02_Table_S2.pdf]

Table S2. Post-hoc baseline-adjusted sensitivity analyses (ANCOVA) for the primary outcome and all 21 secondary outcomes.

Outcome	Unrestricted, adjusted mean (95% CI)	Restricted, adjusted mean (95% CI)	Control, adjusted mean (95% CI)	F(2,53)	p	partial η²	Unrestricted – Restricted, adj. diff (95% CI); p	Unrestricted – Control, adj. diff (95% CI); p	Restricted – Control, adj. diff (95% CI); p
Oswestry Disability Index, %	5.28 (4.43–6.14)	5.18 (4.31–6.05)	6.12 (5.30–6.94)	1.539	.224	.055	0.10 (-1.41 to 1.62); p = 1.000	-0.84 (-2.31 to 0.63); p = .496	-0.94 (-2.41 to 0.53); p = .357
PPT paraspinal (L1–L3), right, kgf/cm²	9.24 (8.84–9.64)	9.33 (8.92–9.75)	9.54 (9.14–9.93)	0.578	.564	.021	-0.09 (-0.81 to 0.62); p = 1.000	-0.30 (-0.99 to 0.40); p = .896	-0.20 (-0.91 to 0.50); p = 1.000
PPT paraspinal (L1–L3), left, kgf/cm²	9.55 (9.04–10.05)	9.54 (9.02–10.06)	8.73 (8.24–9.23)	3.525	.037	.117	0.01 (-0.88 to 0.90); p = 1.000	0.81 (-0.05 to 1.68); p = .073	0.80 (-0.08 to 1.69); p = .087
PPT iliac, right, kgf/cm²	8.93 (8.50–9.35)	9.32 (8.87–9.76)	8.77 (8.36–9.18)	1.646	.203	.058	-0.39 (-1.15 to 0.37); p = .636	0.15 (-0.57 to 0.88); p = 1.000	0.54 (-0.21 to 1.30); p = .242
PPT iliac, left, kgf/cm²	8.80 (8.36–9.24)	9.59 (9.14–10.05)	8.48 (8.06–8.91)	6.709	.003	.202	-0.80 (-1.58 to -0.02); p = .044	0.32 (-0.44 to 1.07); p = .912	1.11 (0.35 to 1.88); p = .002
PPT gluteus medius, right, kgf/cm²	10.20 (9.86–10.54)	10.46 (10.12–10.81)	10.37 (10.04–10.70)	0.635	.534	.023	-0.27 (-0.87 to 0.33); p = .819	-0.17 (-0.76 to 0.41); p = 1.000	0.09 (-0.50 to 0.69); p = 1.000
PPT gluteus medius, left, kgf/cm²	10.14 (9.73–10.55)	10.43 (10.00–10.85)	10.01 (9.60–10.41)	1.082	.346	.039	-0.29 (-1.02 to 0.44); p = 1.000	0.13 (-0.58 to 0.85); p = 1.000	0.42 (-0.30 to 1.15); p = .461
PPT hamstrings, right, kgf/cm²	9.18 (8.81–9.55)	9.30 (8.93–9.68)	9.63 (9.27–9.99)	1.624	.207	.058	-0.12 (-0.77 to 0.52); p = 1.000	-0.45 (-1.09 to 0.19); p = .259	-0.33 (-0.97 to 0.32); p = .658
PPT hamstrings, left, kgf/cm²	9.81 (9.37–10.24)	9.40 (8.95–9.85)	8.67 (8.24–9.09)	7.234	.002	.214	0.41 (-0.37 to 1.19); p = .599	1.14 (0.39 to 1.89); p = .001	0.73 (-0.04 to 1.50); p = .066
PPT knee, right, kgf/cm²	8.31 (7.88–8.74)	8.38 (7.94–8.82)	8.35 (7.94–8.77)	0.025	.975	.001	-0.07 (-0.83 to 0.69); p = 1.000	-0.04 (-0.78 to 0.70); p = 1.000	0.02 (-0.72 to 0.77); p = 1.000
PPT knee, left, kgf/cm²	8.73 (8.20–9.26)	8.32 (7.77–8.86)	8.22 (7.71–8.74)	1.021	.367	.037	0.41 (-0.53 to 1.35); p = .853	0.50 (-0.41 to 1.42); p = .542	0.09 (-0.83 to 1.01); p = 1.000
PPT greater trochanter, right, kgf/cm²	10.07 (9.76–10.38)	10.15 (9.84–10.47)	10.05 (9.75–10.34)	0.137	.873	.005	-0.08 (-0.63 to 0.46); p = 1.000	0.02 (-0.51 to 0.55); p = 1.000	0.11 (-0.43 to 0.64); p = 1.000
PPT greater trochanter, left, kgf/cm²	10.05 (9.78–10.32)	9.96 (9.68–10.24)	9.83 (9.57–10.10)	0.662	.520	.024	0.09 (-0.39 to 0.57); p = 1.000	0.22 (-0.25 to 0.68); p = .773	0.13 (-0.35 to 0.60); p = 1.000
YBT right anterior, cm	67.19 (65.61–68.77)	68.71 (67.08–70.34)	68.08 (66.51–69.65)	0.922	.404	.034	-1.52 (-4.31 to 1.27); p = .550	-0.89 (-3.66 to 1.88); p = 1.000	0.63 (-2.19 to 3.45); p = 1.000
YBT left anterior, cm	67.52 (65.37–69.68)	68.45 (66.20–70.69)	64.60 (62.42–66.78)	3.142	.051	.106	-0.92 (-4.72 to 2.87); p = 1.000	2.93 (-0.92 to 6.77); p = .197	3.85 (-0.11 to 7.81); p = .060
YBT right posterolateral, cm	72.04 (70.13–73.95)	71.94 (69.99–73.89)	69.96 (68.10–71.83)	1.538	.224	.055	0.10 (-3.27 to 3.46); p = 1.000	2.08 (-1.24 to 5.40); p = .381	1.98 (-1.35 to 5.31); p = .440
YBT left posterolateral, cm	73.57 (71.52–75.62)	74.19 (72.10–76.29)	71.64 (69.62–73.65)	1.690	.194	.060	-0.62 (-4.23 to 2.99); p = 1.000	1.93 (-1.64 to 5.51); p = .561	2.56 (-1.04 to 6.15); p = .254
YBT right posteromedial, cm	75.03 (73.09–76.96)	75.05 (73.06–77.04)	70.53 (68.66–72.40)	7.554	.001	.222	-0.02 (-3.47 to 3.42); p = 1.000	4.50 (1.17 to 7.82); p = .005	4.52 (1.16 to 7.88); p = .005
YBT left posteromedial, cm	72.98 (71.33–74.63)	72.71 (71.01–74.41)	71.83 (70.23–73.44)	0.547	.582	.020	0.27 (-2.65 to 3.19); p = 1.000	1.15 (-1.69 to 3.98); p = .967	0.88 (-2.01 to 3.76); p = 1.000
Modified Borg (assessment-level), 0–10	2.08 (1.73–2.44)	1.96 (1.60–2.33)	2.10 (1.76–2.45)	0.172	.843	.006	0.12 (-0.51 to 0.75); p = 1.000	-0.02 (-0.63 to 0.59); p = 1.000	-0.14 (-0.76 to 0.48); p = 1.000
SJ flight time, ms	516.25 (501.54–530.96)	511.00 (495.95–526.06)	485.34 (471.03–499.64)	5.239	.008	.165	5.25 (-20.73 to 31.22); p = 1.000	30.91 (5.56 to 56.27); p = .012	25.67 (0.10 to 51.24); p = .049
CMJ flight time, ms	545.91 (531.47–560.35)	551.31 (536.51–566.11)	517.85 (503.69–532.00)	6.218	.004	.190	-5.40 (-30.82 to 20.03); p = 1.000	28.06 (2.99 to 53.14); p = .023	33.46 (8.12 to 58.80); p = .006

ANCOVA per outcome: follow-up score as dependent variable, baseline score as covariate, randomized group as fixed factor (available-case set, n = 57). Adjusted (marginal) means evaluated at the grand covariate mean, with 95% CIs. Pairwise differences with Bonferroni-adjusted p values and simultaneous 95% CIs. These analyses were not prespecified and are reported as post-hoc sensitivity analyses (Section 2.7); the Bonferroni reference threshold across the 21 secondary outcomes is $p < .0024$. The homogeneity-of-regression-slopes assumption held for all outcomes except PPT knee right (group × baseline interaction $p = .044$), for which the ANCOVA estimate is considered unreliable and no inference is drawn (Section 3.10). PPT, pressure pain threshold; YBT, Y Balance Test; SJ, squat jump; CMJ, countermovement jump.
