# Supplementary material for: Unrestricted Versus Restricted Anterior Knee Displacement During Bodyweight Squat Training in Physically Active Young Adults: An Exploratory Three-Arm Randomized Controlled Trial
Source: J Clin Med. 2026 Sep 15;15(18):7162. doi: 10.3390/jcm15187162 (PMC13607129; doi:10.3390/jcm15187162)
Supplement: Supplementary file 1 [file jcm-15-07162-s001.zip › 03_Supplementary_File_S1_Original_Registration.pdf]

Supplementary File S1 — Original ClinicalTrials.gov Registration Record

NCT06843785 — Version 0 (initial registration)

Provenance. Content reproduced from the archived initial version (Version 0) of record NCT06843785 in the ClinicalTrials.gov study record history, retrieved on 14 August 2026. The authoritative archived record can be inspected directly at **https://clinicaltrials.gov/study/NCT06843785?tab=history**. This file is provided so that the originally registered design and outcome list can be compared with the published report; differences are itemized and explained in Section 2.1 of the manuscript.

Identification and dates

**Registry**	ClinicalTrials.gov, U.S. National Library of Medicine
**NCT number**	NCT06843785
**Sponsor study ID**	Uskudar23
**Brief title**	Comparison of the Effectiveness of Two Different Squat Exercises in Healthy Individuals
**Official title**	Effects of Two Different Squat Exercise Patterns on Muscle Strength, Performance, Low Back Pain and Balance in Young Adult Healthy Individuals
**Record submitted**	19 February 2025
**Record first posted**	25 February 2025
**Recruitment status at registration**	Not yet recruiting (record verified February 2025)
**Estimated study start**	1 March 2025
**Estimated primary completion**	1 May 2025
**Estimated study completion**	15 May 2025
**Lead sponsor**	Uskudar University
**Principal investigator**	Dr. Ömer Şevgin, Assistant Professor, Uskudar University
**Study chair**	Amirkiya Movahhedi, Bahçeşehir University

Brief summary (as registered)

The research aims to examine how two distinct squat variations — allowing versus restricting knee movement beyond toe position — affect lower back pain, muscle strength, and balance outcomes.

Detailed description (as registered)

Sixty volunteers aged 18–30 with active physical activity levels will be randomized into three groups receiving either a squat program allowing forward knee translation (n=20), restricted knee movement using a wooden board barrier (n=20), or no intervention (n=20). Both exercise groups will perform one set of 20 repetitions with 30-second contraction/rest cycles, three times weekly for six weeks through telerehabilitation supervision. Pre- and post-intervention assessments will measure back pain, knee pain, muscle thickness, strength, function, and balance.

Study design (as registered)

**Study type**	Interventional

**Allocation**	Randomized (described as computer system and closed-envelope method)
**Intervention model**	Parallel assignment, three arms
**Masking**	Triple (participant, investigator, outcomes assessor) — *declared in the manuscript (Section 2.1) as entered in error; no party was blinded*
**Primary purpose**	Treatment
**Estimated enrollment**	60

Arms and interventions (as registered)

Arm	Description
**Knee-past-toes squat** (experimental, n=20)	Feet pelvis-width apart; participants squat to their most comfortable point, looking straight ahead, without heels leaving the floor; arms extended forward; hip-to-floor distance measured vertically; three trials averaged.
**Knee-not-past-toes squat** (experimental, n=20)	A wooden board placed just distal to the first toe of each foot restricts anterior knee movement; squat to 90° knee flexion with the same positioning; knee angle confirmed with a goniometer.
**Control** (n=20)	Participants will not be subjected to any intervention and will not be asked to perform any action.

Outcome measures (as registered)

Primary outcomes (three co-primary measures; time frame 8 weeks):

- **Oswestry Disability Index** — functional loss from low back pain across 10 categories; scored 0–100, higher scores indicating greater disability.
- **Y Balance Test** — dynamic balance and postural stability; anterior, posteromedial, and posterolateral reach normalized by leg length; composite calculated from all directions.
- **International Physical Activity Questionnaire – Short Form (IPAQ-SF)** — physical activity across work, transportation, household/gardening, and leisure domains; MET-based score with three activity categories.

Secondary outcomes: none listed in the registry outcome fields.

Eligibility criteria (as registered)

Inclusion	Exclusion
• Ages 18–30<br>• Physically active per IPAQ-SF<br>• No psychological, neurological, orthopedic, or rheumatic disorders<br>• No lower-extremity fractures or surgery within the past year<br>• Able to perform a traditional squat<br>• Turkish language fluency	• Pregnancy or suspected pregnancy<br>• Knee valgus diagnosis<br>• Pes planus/cavus or hip rotation diagnoses<br>• Strength asymmetry between limbs<br>• Acute knee osteoarthritis history<br>• Regular NSAID or disease-modifying drug use within the past month<br>• Intra-articular injection within 6 months<br>• Contraindicated comorbidities (advanced osteoporosis, vertigo, neurological disease)

Subsequent registry versions (change history)

Version	Submitted	Modules changed
0	19 Feb 2025	Initial registration (not yet recruiting)
1	14 Mar 2025	Study status; contacts/locations (recruiting)
2	11 Jun 2025	Status (completed); study design; location information
3	23 Jul 2026	Sponsor/collaborator details; outcome measurement specifications
4	11 Aug 2026	Comprehensive correcting amendment across 8 modules (identification, description, design, interventions, eligibility, outcomes, data sharing), aligning the record with the conduct of the trial as declared in Section 2.1 of the manuscript
