# Supplementary material for: Unrestricted Versus Restricted Anterior Knee Displacement During Bodyweight Squat Training in Physically Active Young Adults: An Exploratory Three-Arm Randomized Controlled Trial
Source: J Clin Med. 2026 Sep 15;15(18):7162. doi: 10.3390/jcm15187162 (PMC13607129; doi:10.3390/jcm15187162)
Supplement: Supplementary file 1 [file jcm-15-07162-s001.zip › 04_Supplementary_File_S2_Verification_Report.pdf]

Supplementary File S2 — Verification Report

Manuscript jcm-4500801 — item-by-item verification of all reported statistics against the participant-level dataset

Purpose. In response to the editorial request for supporting data and documentation, every descriptive and inferential statistic reported in Tables 2–5 of the manuscript, and all values in Tables S1–S2, were re-computed from the anonymized participant-level dataset submitted as Supplementary Data S1, using the analysis code submitted as Supplementary Code S1. This report documents the method, the result of the comparison, the discrepancies found and corrected (marked in red in the revised manuscript), and the documentary basis for the intervention schedule, the trial-registration information, and the withdrawal of the IKDC outcome. Prepared August 2026.

1. Dataset

The dataset contains one row per participant in the available-case analysis set: n = 57 (unrestricted 19, restricted 18, control 20), matching Section 3.1 and Figure 1. It includes baseline and week-6 values for the primary outcome and all 21 secondary outcomes, plus the withdrawn IKDC entries exactly as recorded. No identifying information is included. The Oswestry column holds the raw 0–50 score; the manuscript reports the doubled percentage, which leaves every test statistic unchanged.

2. Verification method and overall result

All 132 group-level mean ± SD cells of Tables 2–4, all 22 ANOVA F/p/η^2 entries, the within-group p values and effect sizes, and the pairwise differences with 95% CIs (Table 5, Table S1) were re-computed and compared cell by cell. Representative exact reproductions include: Oswestry $F(2,54) = 1.999$, $p = .145$, $\eta^2 = 0.069$, largest pairwise difference −2.9 percentage points (95% CI −5.9 to 0.0); left-hamstring PPT $F(2,54) = 3.495$, $p = .037$, $\eta^2 = 0.115$; SJ flight time $F = 0.380$, $p = .686$; CMJ $F = 0.092$, $p = .912$; all six Y Balance Test directions; all Borg values. The ANCOVA results of Table S2 (e.g., Oswestry adjusted $F(2,53) = 1.539$, $p = .224$; left-hamstring PPT $F = 7.234$, $p = .002$; right posteromedial YBT $F = 7.554$, $p = .001$) derive from the same dataset and are fully reproduced by Supplementary Code S1.

2b. How to verify each disputed item directly (reviewer guide)

Disputed item	Verification path (typical time)
All Table 2–5 statistics	Run Supplementary Code S1 on Supplementary Data S1 (Python 3; ~1 min), or read the pre-computed output in Supplementary Code S1 Output Log and compare against the tables. (5 min)
ANCOVA results (Table S2, Section 3.10)	Same script, "ANCOVA" lines per outcome. (5 min)
IKDC withdrawal	Open Data S1, columns ikdc_pre/post_as_recorded: integers 68–73 only; compare with the normative distribution cited in §4. (2 min)
Table 1 demographics	Data S1 columns sex, age_years, weight_kg, height_cm; group summaries reproduce Table 1. (3 min)
Registration dates and original outcome list	Public archive: https://clinicaltrials.gov/study/NCT06843785?tab=history — Version 0 (submitted 19 Feb 2025); cf. Supplementary File S1. (5 min)
Intervention schedule (18 sessions)	Version 0 Detailed Description: "three times weekly for six weeks"; §5 of this report. (2 min)
Origin of the corrected Table 2 cells	§7 of this report: the three erroneous values appear in the source thesis tables and do not exist in the dataset. (3 min)

3. Discrepancies identified and corrected (red in the revised manuscript)

Location	Item	Previously reported	Corrected (from dataset)	Consequence
Table 2, gluteus medius right, restricted	follow-up SD	10.51 ± 1.24	**10.51 ± 0.93**	none (descriptive)

Table 2, gluteus medius left, restricted	baseline mean; derived d	10.21 ± 1.34; d = 0.17	**10.14 ± 1.34; d = 0.23**	none (within-group p = .413 unchanged)
Table 2, greater trochanter left, restricted	follow-up mean ± SD; derived d	10.39 ± 1.39; d = 0.26	**10.14 ± 1.37; d = 0.10**	none (within-group p = .365 unchanged)
Table 2, paraspinal left	η^2	0.050	**0.051**	none
Table 2, iliac right	η^2	0.006	**0.007**	none
Table 2, gluteus medius right	η^2	0.011	**0.014**	none
Table 2, greater trochanter left	η^2	0.059	**0.036**	none
Table 3, YBT right posterolateral	F	0.278	**0.248**	none (p = .781, η^2 = 0.009 were already correct)
Section 3.4 (text)	η^2 range of remaining comparisons	0.001–0.059	**0.001–0.054**	none
Section 3.5 (text)	trochanter ceiling-proximity range	10.04–10.09	**9.95–10.09**	none
Sections 2.6.1 and 3.5 (text)	ceiling-proximity claim	"within 8%" of ceiling	**"within 10%" of ceiling**	none

No corrected value changes any inference: every affected comparison remains non-significant, and no conclusion of the manuscript depends on any corrected cell. All other verified values were identical to those reported (differences of ±0.01 attributable to rounding convention were not treated as errors).

4. Basis for the withdrawal of the IKDC outcome

The dataset contains the IKDC entries exactly as recorded: whole numbers between 68 and 73 only, at both time points, in all three groups.

**Recorded value**	68	69	70	71	72	73
**Baseline (n = 57)**	1	4	10	12	16	14
**Follow-up (n = 57)**	0	1	6	13	14	23

The standard 2000 IKDC subjective score is a transformation — (raw score − 18)/87 × 100 — that rarely yields integers and, in healthy active adults aged 18–24, has normative mean ≈ 86–89 with SD ≈ 16–19 (Anderson et al., 2006). A distribution confined to six integer values (68–73) is not compatible with correctly transformed scores, and the underlying item-level forms could not be retrieved to re-derive them. The outcome was therefore withdrawn from the manuscript in its entirety (Sections 2.1, 2.6.3, 4.6), and these columns are retained in Supplementary Data S1 solely so that the basis for the withdrawal can be inspected directly.

5. Documentary basis for the 18-session intervention schedule

Three sources bear on the session count. (i) The original February 2025 registration (Supplementary File S1, Detailed Description) specifies "three times weekly for six weeks" — arithmetically 18 sessions — and predates enrolment and both manuscript versions. (ii) The narrative protocol description of the source master's thesis likewise states six weeks at three sessions per week. (iii) A protocol table in the same thesis lists sessions only to "13–15", which conflicts with the thesis's own narrative description and with the registry; the "15 sessions / 540 repetitions" wording of the original submission reproduced this table. The delivered protocol was 18 sessions (720 repetitions), consistent with sources (i) and (ii) and confirmed by all authors; real-time attendance verification at every session is described in Section 2.5. The corrected count is also the only one consistent with the progression arithmetic stated in Section 2.5 (3 × 20 + 6 × 30 + 3 × 40 + 6 × 60 = 720).

6. Trial-registration information

Version	Submitted	Status / modules changed
0	19 Feb 2025	Initial registration; not yet recruiting (posted 25 Feb 2025; first enrolment 1 Mar 2025 → prospective)
1	14 Mar 2025	Status, contacts/locations (recruiting)
2	11 Jun 2025	Status (completed); design; location
3	23 Jul 2026	Sponsor/collaborator details; outcome measurement specifications
4	11 Aug 2026	Comprehensive correcting amendment (8 modules), aligning the record with the conduct declared in Section 2.1

The full history is permanently public at https://clinicaltrials.gov/study/NCT06843785?tab=history; the original version is reproduced as Supplementary File S1. The two dates previously cited in different manuscript versions (19 and 25 February 2025) are the submission and first-posting dates of the same original record; both precede first enrolment.

7. Correspondence between the source thesis, the dataset, and the manuscript

Because the trial derives from a publicly deposited master's thesis, the three documents can be cross-checked. All 173 mean ± SD statistics extractable from the thesis text were compared automatically against every group × time-point summary computable from Supplementary Data S1. The result: **every outcome statistic reported in the thesis reproduces from the dataset**, including the baseline demographics now added to Data S1 (e.g., weight 73.32 ± 14.38 / 73.06 ± 13.87 / 72.35 ± 16.82 kg) and the withdrawn IKDC values (71.28 ± 1.57, etc.). The only thesis values that do not correspond to the dataset are: (i) jump height and power outputs of the My Jump 2 application, which are algebraic transformations of flight time and were specified as not analysed (Section 2.6.4); and (ii) exactly the three erroneous Table 2 values corrected in this revision (10.51 ± 1.24; 10.21 ± 1.34; 10.39 ± 1.39), which appear in the thesis tables but in no computation from the dataset — establishing that these were transcription errors originating in the thesis tables and carried into the manuscript, not values of the underlying data. This closes the verification triangle: thesis ↔ dataset ↔ manuscript.
