# Supplementary material for: Unrestricted Versus Restricted Anterior Knee Displacement During Bodyweight Squat Training in Physically Active Young Adults: An Exploratory Three-Arm Randomized Controlled Trial
Source: J Clin Med. 2026 Sep 15;15(18):7162. doi: 10.3390/jcm15187162 (PMC13607129; doi:10.3390/jcm15187162)
Supplement: Supplementary file 1 [file jcm-15-07162-s001.zip › 08_Supplementary_File_S3_CONSORT_2025_Checklist.pdf]

CONSORT 2025 Checklist of Information to Include When Reporting a Randomised Trial

Manuscript: Unrestricted versus Restricted Anterior Knee Displacement during Bodyweight Squat Training in Physically Active Young Adults: An Exploratory Three-Arm Randomized Controlled Trial (revision 2)

Locations refer to numbered sections, tables, and figures of the revised manuscript. Status: ✓ reported · ◐ partially reported · ✗ not reported · — not applicable. Reporting also follows the CONSORT extension for nonpharmacologic treatments. This checklist replaces the CONSORT 2010 checklist submitted with the original manuscript.

Item	Checklist item (CONSORT 2025)	Status	Location	Comment
**Title and abstract**				
**1a**	Identification as a randomised trial	✓ Reported	Title (p. 1)	Title: "…An Exploratory Three-Arm Randomized Controlled Trial"
**1b**	Structured summary of the trial design, methods, results, and conclusions	✓ Reported	Abstract (p. 1)	Structured Background/Objectives – Methods – Results – Conclusions; exploratory framing and unadjusted comparisons stated
**Open science**				
**2**	Name of trial registry, identifying number (with URL), and date of registration	✓ Reported	Abstract; Section 2.1	ClinicalTrials.gov NCT06843785 (https://clinicaltrials.gov/study/NCT06843785); record submitted 19 February 2025 and first posted 25 February 2025, before first enrolment on 1 March 2025
**3**	Where the trial protocol and statistical analysis plan can be accessed	◐ Partially reported	Section 2.1; Data Availability Statement	Registered protocol summary at ClinicalTrials.gov; original registration provided as Supplementary File 1; access statement for the ethics-approved protocol is being finalized in this revision
**4**	Where and how the individual deidentified participant data (including data dictionary), statistical code, and any other materials can be accessed	✓ Reported	Data Availability Statement	Anonymized participant-level dataset available from the corresponding author on reasonable request
**5a**	Sources of funding and other support, and role of funders	✓ Reported	Funding (back matter)	"This research received no external funding."
**5b**	Financial and other conflicts of interest of the manuscript authors	✓ Reported	Conflicts of Interest (back matter)	"The authors declare no conflicts of interest."
**Introduction**				
**6**	Scientific background and rationale	✓ Reported	Section 1	Acute-biomechanics literature and rationale for a training-study comparison
**7**	Specific objectives related to benefits and harms	✓ Reported	Section 1 (final paragraph); Section 2.5	Exploratory objectives; no directional hypothesis; pain/adverse-event monitoring with protocol-specified 5/10 VAS withdrawal threshold
**Methods**				
**8**	Details of patient or public involvement in the design, conduct, and reporting of the trial	✓ Reported	Section 2.1	No patient or public involvement
**9**	Description of trial design, including type of trial, allocation ratio, and framework	✓ Reported	Section 2.1; Abstract	Single-centre, three-arm, parallel-group, 1:1:1; exploratory framework stated explicitly
**10**	Important changes to the trial after it commenced, including any outcomes or analyses that were not prespecified, with reason	✓ Reported	Section 2.1	All differences between the registry record and this report declared itemwise; outcome reclassification at analysis stage declared as not prespecified; August 2026 registry amendment described; the IKDC

Item	Checklist item (CONSORT 2025)	Status	Location	Comment
				outcome was withdrawn during this revision because its scoring could not be verified against source records (Sections 2.1, 2.6.3, 4.6)
**11**	Settings and locations where the trial was conducted	✓ Reported	Sections 2.1, 2.5	Single site: face-to-face baseline and follow-up assessments at the physiotherapy assessment laboratory of Bahçeşehir University, Istanbul, Türkiye (Section 2.1); intervention delivered at participants' homes by synchronous telerehabilitation; ethics approval and sponsorship by Üsküdar University
**12a**	Eligibility criteria for participants	✓ Reported	Section 2.2	Inclusion and exclusion criteria; IPAQ-SF used for eligibility
**12b**	If applicable, eligibility criteria for sites and for individuals delivering the interventions	◐ Partially reported	Section 2.5	All sessions delivered by a single qualified physiotherapist (A.M.); years of clinical experience explicitly not reported
**13**	Intervention and comparator with sufficient details to allow replication	✓ Reported	Section 2.5	18-session progressive schedule, tempo (metronome 60 bpm), work–rest structure, positioning, board specification, supervision mode; cumulative 720 repetitions
**14**	Prespecified primary and secondary outcomes, including measurement variable, analysis metric, method of aggregation, and time point	✓ Reported	Sections 2.1, 2.6	Measures, metrics and time points reported; three unregistered measures declared as specified in the thesis protocol but absent from the registry outcome fields (interpreted as exploratory); the IKDC outcome withdrawn because its scoring could not be verified
**15**	How harms were defined and assessed	✓ Reported	Sections 2.5, 2.6, 3.9	Nonsystematic: protocol-specified 5/10 VAS pain withdrawal threshold; spontaneously reported adverse events recorded at sessions and visits
**16a**	How sample size was determined, including all assumptions	✓ Reported	Section 2.3	G*Power 3.1.9.7, one-way ANOVA, effect size 0.799, $\alpha = .05$, power .90 → 20/group; anchoring and its consequences discussed; minimum detectable effect with $n = 57$ reported ($\eta^2 \approx 0.15$)
**16b**	Explanation of any interim analyses and stopping guidelines	✓ Reported	Section 2.3	No interim analysis planned or performed; no formal stopping guideline
**17a**	Who generated the random allocation sequence and the method used	✓ Reported	Section 2.4	Independent researcher not otherwise involved; random-number functions of Microsoft Excel
**17b**	Type of randomisation and details of any restriction	✓ Reported	Section 2.4	Restricted randomization: pre-generated list of fixed composition (20 entries per group), randomly permuted; no blocking or stratification
**18**	Mechanism used to implement the random allocation sequence, describing any steps to conceal the sequence	✓ Reported	Section 2.4	List prepared and held by a person outside the research team, inaccessible to the enrolling investigator; envelopes not used (registry description declared as an error)
**19**	Whether the personnel who enrolled and those who assigned participants had access to the random allocation sequence	✓ Reported	Section 2.4	Enrolment by A.M. without access to the list; assignment executed sequentially by the independent list-holder
**20a**	Who was blinded after assignment to interventions	✓ Reported	Section 2.4	No party blinded (participants, treating physiotherapist, outcome assessors); registry "triple masked" entry declared incorrect
**20b**	If blinded, how blinding was achieved and description of the similarity of interventions	— Not applicable	Section 2.4	Not applicable: interventions visibly different; no blinding attempted
**21a**	Statistical methods used to compare groups for primary and secondary outcomes, including harms	✓ Reported	Section 2.7	One-way ANOVA on follow-up scores; Tukey HSD / Games-Howell; harms summarized descriptively; Bonferroni reference threshold for 22 exploratory outcomes

Item	Checklist item (CONSORT 2025)	Status	Location	Comment
**21b**	Definition of who is included in each analysis and in which group	✓ Reported	Section 2.7; Section 3.1	Available-case (complete-case) analysis of 57 completers, analysed by randomized group; explicitly distinguished from intention-to-treat
**21c**	How missing data were handled in the analysis	✓ Reported	Sections 2.7, 3.1	No imputation and no missing-data sensitivity analysis; declared and listed as a limitation
**21d**	Methods for any additional analyses, distinguishing prespecified from post hoc	✓ Reported	Sections 2.7, 3.10	Post-hoc baseline-adjusted (ANCOVA) sensitivity analyses performed for all outcomes and labelled post hoc (Sections 2.7, 3.10; Table S2)
**Results**				
**22a**	For each group, the numbers randomly assigned, receiving intended intervention, and analysed for the primary outcome	✓ Reported	Section 3.1; Figure 1	60 randomized (20/20/20); 57 analysed (19/18/20)
**22b**	For each group, losses and exclusions after randomisation, together with reasons	✓ Reported	Section 3.1; Figure 1	Three discontinuations (1/2/0) during sessions 1–5, personal reasons or time constraints; none for adverse events or pain
**23a**	Dates defining the periods of recruitment and follow-up	✓ Reported	Section 2.1	March 2025 to May 2025
**23b**	If relevant, why the trial ended or was stopped	✓ Reported	Section 2.1	Ended as planned when the target sample completed the protocol
**24a**	Intervention and comparator as actually administered (delivery, adherence, fidelity)	✓ Reported	Sections 2.5, 3.1	Attendance verified in real time by the supervising physiotherapist; all 37 exercise-arm completers attended all 18 sessions (100% session adherence among completers); fixed progression; technique errors corrected but not counted
**24b**	Concomitant care received during the trial for each group	◐ Partially reported	Section 2.5	Participants asked not to begin new structured exercise; habitual activity not monitored quantitatively after baseline (acknowledged limitation)
**25**	A table showing baseline demographic and clinical characteristics for each group	✓ Reported	Table 1; Section 3.2	Descriptive presentation; baseline significance testing avoided, one visible imbalance handled as confounded
**26**	For each primary and secondary outcome, by group: numbers analysed, results, effect size and precision	✓ Reported	Sections 3.3–3.8; Tables 2–5; Table S1	Primary outcome with all pairwise 95% CIs; the 21 secondary outcomes with F, p, η^2 and 95% CIs in Table S1; no binary outcomes; Table S1 regenerated from the participant-level dataset and included; adjusted estimates in Table S2
**27**	All harms or unintended events in each group	✓ Reported	Section 3.9	No adverse events, injuries, or pain above the withdrawal threshold in any arm
**28**	Any other analyses performed, including subgroup and sensitivity analyses, distinguishing prespecified from post hoc	✓ Reported	Sections 2.7, 3	No subgroup analyses; post-hoc baseline-adjusted sensitivity analyses reported in Section 3.10 and Table S2, distinguished as post hoc
**Discussion**				
**29**	Interpretation consistent with results, balancing benefits and harms, and considering other relevant evidence	✓ Reported	Sections 4.1–4.5	Cautious exploratory interpretation; control-group changes, floor effect, and acute-biomechanics literature integrated; prescriptive claims withdrawn
**30**	Trial limitations, addressing sources of potential bias, imprecision, generalisability, and multiplicity	✓ Reported	Section 4.6 (and 2.3, 2.7)	Unblinded design, available-case analysis, unadjusted comparisons, floor effect, censored algometry ceiling, multiplicity, generalisability limits

Reference: Hopewell S, Chan A-W, Collins GS, et al. CONSORT 2025 statement: updated guideline for reporting randomised trials. BMJ 2025;389:e081123 (co-published in Lancet, JAMA, Nat Med, PLOS Med). Nonpharmacologic reporting also follows Boutron I, et al. Ann Intern Med 2017;167:40–47.
